# Slam protein dictates subcellular localization and translation of its own mRNA

**DOI:** 10.1371/journal.pbio.2003315

**Published:** 2017-12-04

**Authors:** Shuling Yan, Sreemukta Acharya, Stephanie Gröning, Jörg Großhans

**Affiliations:** Institute for Developmental Biochemistry, Medical School, University of Göttingen, Göttingen, Germany; MRC Laboratory of Molecular Biology, United Kingdom of Great Britain and Northern Ireland

## Abstract

Many mRNAs specifically localize within the cytoplasm and are present in RNA-protein complexes. It is generally assumed that localization and complex formation of these RNAs are controlled by trans-acting proteins encoded by genes different than the RNAs themselves. Here, we analyze *slow as molasses* (*slam*) mRNA that prominently colocalizes with its encoded protein at the basal cortical compartment during cellularization. The functional implications of this striking colocalization have been unknown. Here, we show that *slam* mRNA translation is spatiotemporally controlled. We found that translation was largely restricted to the onset of cellularization when Slam protein levels at the basal domain sharply increase. *slam* mRNA was translated locally, at least partially, as not yet translated mRNA transiently accumulated at the basal region. *Slam* RNA accumulated at the basal domain only if Slam protein was present. Furthermore, a *slam* RNA with impaired localization but full coding capacity was only weakly translated. We detected a biochemical interaction of *slam* mRNA and protein as demonstrated by specific co-immunoprecipitation from embryonic lysate. The intimate relationship of *slam* mRNA and protein may constitute a positive feedback loop that facilitates and controls timely and rapid accumulation of Slam protein at the prospective basal region.

## Author summary

While proteins and their encoding messenger RNAs share the same intracellular space during the translation process, thereafter they are usually spatially and biochemically separated. RNA localization follows a specific subcellular pattern—such as apical or basal—and is thought to have important physiological implications during development, which are generally independent from the protein function. Here, we investigate the potential mutual dependencies between *slam* mRNA and its encoded protein during cellularization in early *Drosophila* embryos. *slam* RNA and protein are known to colocalize and are essential for epithelial compartmentalization and timely invagination of the plasma membrane between adjacent nuclei. We now show that Slam protein is required for RNA localization at the basal domain and that this event is needed for efficient translation. In addition to the functional interactions, we find that *slam* RNA and protein are both present in a specific molecular complex. Our findings indicate that *slam* is locally translated and that the interaction between Slam protein and RNA constitutes a self-enhancing mechanism leading to the fast accumulation of Slam protein at the basal domain during the first minutes of cellularization.

## Introduction

Subcellular RNA localization is a widespread phenomenon [1–4]. A large-scale survey of RNA localization in *Drosophila* embryos by RNA in situ hybridization with fluorescent probes revealed that about 70% of all tested transcripts were distributed in a specific subcellular pattern, such as apical or basal localization [5]. The physiological relevance of RNA localization is unknown for most of these transcripts, however. The function and mechanism of RNA localization have been studied in detail in numerous cases. Specific localization is usually mediated by cis-acting elements within the transcript and trans-acting factors [6–9]. Trans-acting factors are encoded by transcripts different than the localizing RNA, in most cases.

We reported previously that *slow as molasses* (*slam*) RNA and protein strikingly colocalize during early *Drosophila* development [10]. However, the function and underlying mechanism of *slam* mRNA-protein colocalization have so far not been analyzed. *slam* is a key player in the change from syncytial to cellular development [11–14]. *slam* is specifically expressed during this stage and is required for furrow invagination and separation of cortical domains during cellularization and, later, for germ cell migration [10, 15, 16]. *slam* mRNA and protein strongly accumulate at the basal cortical domain, which forms the so-called furrow canal (FC). For the specification of the basal domain, *slam* functions redundantly with *nullo*. Markers of the FC such as Dia are present in *slam* mutants but are uniformly distributed in *slam nullo* double mutants [17].

An important feature of the transition from syncytial to cellular development is the rapid and coordinated change of several processes within a few minutes only. As zygotic gene expression gradually increases during the course of many minutes, additional posttranscriptional mechanisms potentially control the temporally and spatially regulated activity of the key players such as *slam* to ensure a switch-like change in behavior of the cellular processes from nuclear cycle 13 to cycle 14.

Here, we investigate the functional and molecular interactions of *slam* mRNA and protein and address the function of *slam* RNA localization and RNA-protein colocalization. In addition to functional interactions, we identified a specific biochemical interaction, in that Slam protein specifically coprecipitated with *slam* mRNA.

## Results

### Temporal and spatial regulation of Slam protein expression

**Fig 1:**
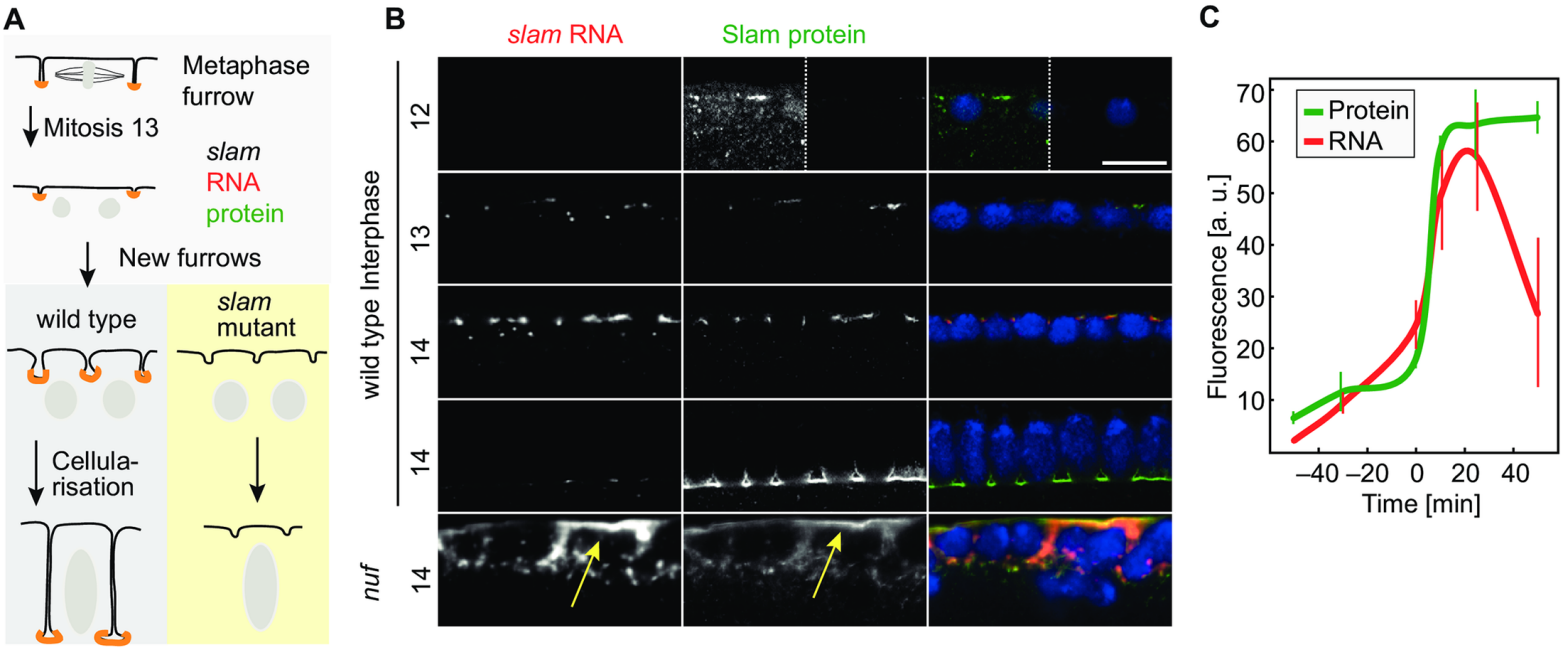
Expression profile of *slam* RNA and protein. **(A)** Schematic drawing of cellularization. *slam* RNA (red) and protein (green) mark the basal cortical domain at the tip of the metaphase furrow and the cellularization furrow. Orange marks RNA protein colocalization. The metaphase furrow transforms into the cellularization furrow. At the onset of cellularization, a new furrow emerges between daughter nuclei and becomes indistinguishable from the old furrows soon after. Furrow invagination is absent in *slam* mutants. **(B)** Fixed wild type and embryos from *nuf* females stained for *slam* RNA (grey/red), Slam protein (grey/green), and DNA (blue). Yellow arrows point to mislocalized *slam* RNA and protein in *nuf* embryos. Insets are images with increased contrast. Scale bars = 10 μm. **(C)** Quantification of fluorescence at the FC after fixation and staining for *slam* RNA and protein. Embryos were manually staged according to morphology, i.e., furrow depth and nuclear shape/length and its corresponding time relative to mitosis 13. Error bars indicate standard error of the mean. *N* = 3 embryos. About 10 furrows were scored in each embryo. The underlying data for this figure can be found in S1 Data. a. u., arbitrary units.

*slam* RNA and protein (about 4,000 nucleotides and 1,173 amino acid residues, S1 Fig) mark the basal region of the cellularization furrow, the FC (Fig 1A and 1B) [10]. Morphologically visible furrows emerge within a few minutes after the last mitosis by invagination of the plasma membrane between adjacent nuclei (Fig 1A). *slam* RNA and protein colocalization was also observed when Slam was ectopically localized (Fig 1B). We achieved ectopic Slam protein localization by employing embryos from *nuf* females, which are impaired for the recycling endosomes [17, 18]. In these embryos, we detected *slam* RNA together with Slam protein at the apical membrane. These data show that *slam* RNA and protein colocalize also in situations of ectopic localization and indicate that the mechanisms controlling *slam* RNA and protein localization are interconnected.

Previous expression and histological analysis indicated an up-regulation of *slam* RNA and protein during the blastoderm stage [10, 15, 16]. Expression analysis by NanoString technology defined the window of strong up-regulation to nuclear cycle 13 and early cellularization (cell cycle 14) [19]. We confirmed these data by measuring total RNA and protein levels. We found an about 10-fold up-regulation of total RNA levels during cellularization (2–3 h) by quantitative PCR (S2A Fig) and a peak of total protein levels in extracts from embryos in cellularization (S2B Fig). Slam is required for formation of the cellularization furrows [15, 16] and identity of the basal domain [10]. As *slam* RNA and protein are strongly enriched at the FC, we next analyzed accumulation of *slam* RNA and protein specifically at the FC in fixed and stained embryos, which were staged by nuclear cycle and by the length of the furrow and nuclei (S2C Fig). By quantification of the fluorescence signal at the FC, we observed a strong (6-fold) up-regulation of both localized RNA and protein within a short period of a few minutes at the transition from mitosis 13 to interphase 14 (Fig 1C). Following this up-regulation, protein levels remained constant, whereas RNA levels gradually decreased after 30 min during the second half of cellularization (Fig 1C).

**Fig 2:**
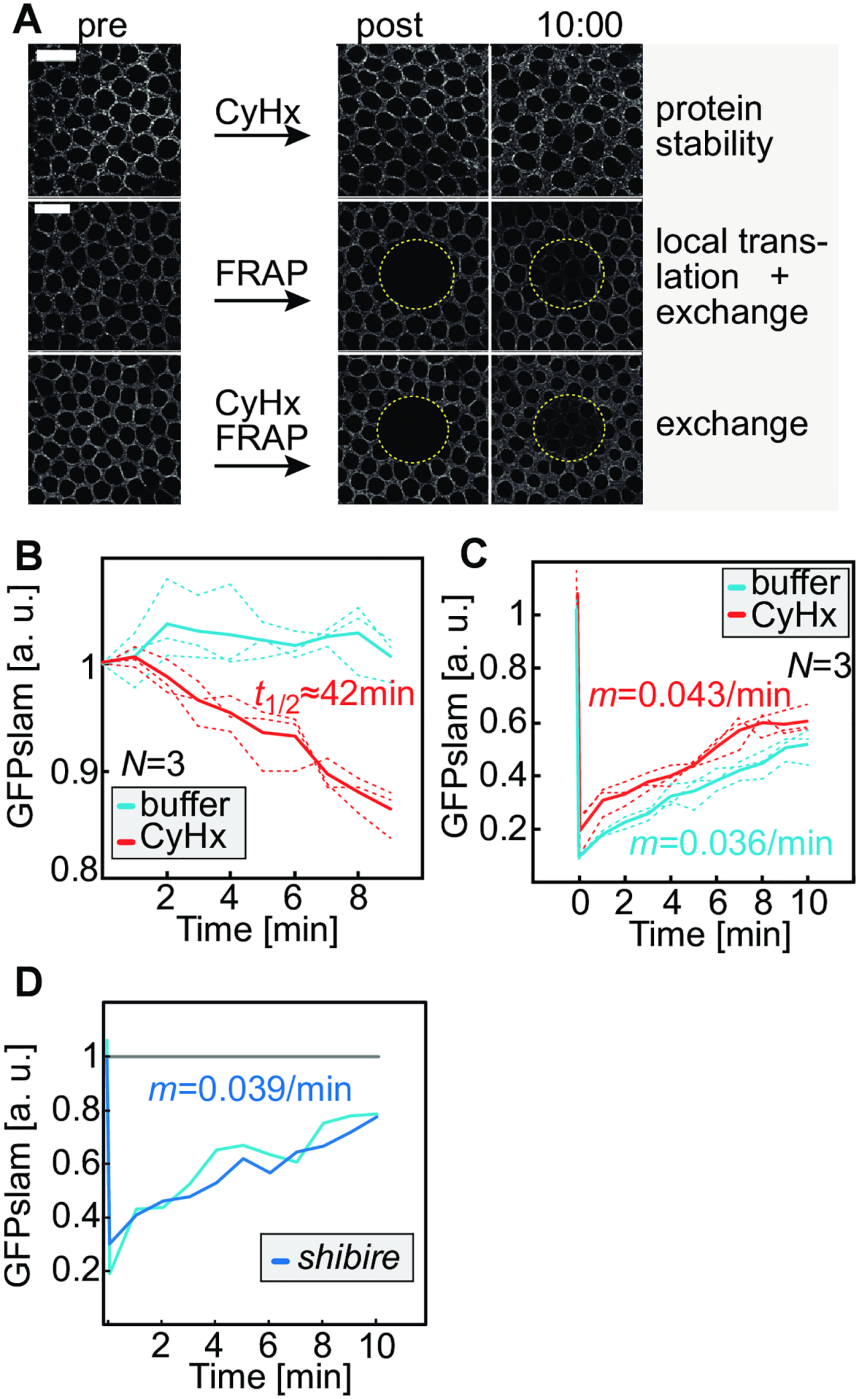
GFP-slam protein turnover and mobility. Wild-type embryos **(A—C)** injected with buffer or CyHx or embryos **(D)** from *shibire* heterozygous females shifted to nonpermissive temperature in mid-cellularization, expressing GFP-slam. **(A)** Images from time-lapse recordings, pre-bleach, post-bleach, and 10 min after bleach. Scale bars = 10 μm. **(B)** Time course of GFP-slam fluorescence following injection of CyHx. Corresponds to upper images in (A). Approximate half-life (*t*_1/2_) of GFP-slam during the measurement period was calculated by assuming an exponential decay. **(C)** Time course of GFP-slam fluorescence following injection and photobleaching within the bleached area (circle in yellow). Corresponds to middle and lower row of images in (A). **(D)** Time course of GFP-slam fluorescence following photobleaching in the bleached area (circle in yellow). **(C, D)** Recovery rate (*m*) was calculated by linear fitting. **(D)** Fluorescent trace was normalized to unbleached area. The underlying data for this figure can be found in S1 Data. a. u., arbitrary units.

Based on the colocalization of RNA and protein, we hypothesized that translation of *slam* might be linked to RNA localization. To address this hypothesis, we first defined Slam protein stability and the timing of translation. *slam* RNA may be translated with a constant rate throughout cellularization or specifically during onset of cellularization. To separate the contribution of translation and degradation to the steady state levels of Slam protein, we first measured the stability of green fluorescent protein (GFP)-slam protein during cellularization (Fig 2A, S1 Fig). GFP-slam serves as a proxy for untagged Slam, because GFP-slam can rescue the cellularization phenotype of *slam* mutants. Furthermore, the dynamics of GFP-slam largely reflect the dynamics of Slam protein [17]. To measure the half-life of GFP-slam, we injected cycloheximide into embryos in cellularization to stop new translation (S3A–S3C Fig) and recorded the persistence of GFP-slam fluorescence. From the measured decay of GFP-slam during periods of 10 min, we extrapolated an estimated half-life of approximately 40 min (Fig 2A and 2B). A half-life of 40 min is in the range of the length of cellularization and thus indicates that GFP-slam is quite a stable protein. Together with the relatively stable expression levels of GFP-slam as well as endogenous Slam protein (Fig 1C, S2B Fig), the long half-life indicates a low translation rate of *slam* mRNA after the initial phase of cellularization. We could not measure the role of GFP-slam synthesis for the dynamics of GFP-slam during the onset of cellularization, as injection of cycloheximide would induce a mitotic arrest.

Secondly, we analyzed the mobility of GFP-slam by quantifying fluorescence recovery after photobleaching (FRAP). Previously, we found that the recovery of GFP-slam fluorescence dramatically changes from fast and complete during the onset of cellularization to slow and incomplete recovery during the course of cellularization [17]. The slow fluorescence recovery after the initial phase of cellularization may be due to the exchange of bleached and unbleached molecules by mobile GFP-slam molecules. Alternatively, fluorescence recovery may be due to translation of new GFP-slam molecules. We distinguished these 2 options by FRAP experiments in embryos, in which new GFP-slam synthesis was blocked by cycloheximide. We observed a comparable recovery rate with and without cycloheximide. Thus, new GFP-slam translation does not contribute to fluorescence recovery during cellularization (Fig 2A and 2C). These data are consistent with the long half-life of GFP-slam during cellularization. Furthermore, the slow exchange of Slam molecules did not require vesicle budding, because a mutation in Dynamin (*shibire*) [20] had little influence on the recovery rate (Fig 2D). In summary, our data suggest that Slam protein is largely synthesized during a short period of a few minutes at the transition from mitosis 13 to interphase 14. Following this initial phase, Slam protein is subject to low turnover during the remainder of cellularization.

### Not yet translated *slam* mRNA transiently accumulates at the prospective basal domain

**Fig 3:**
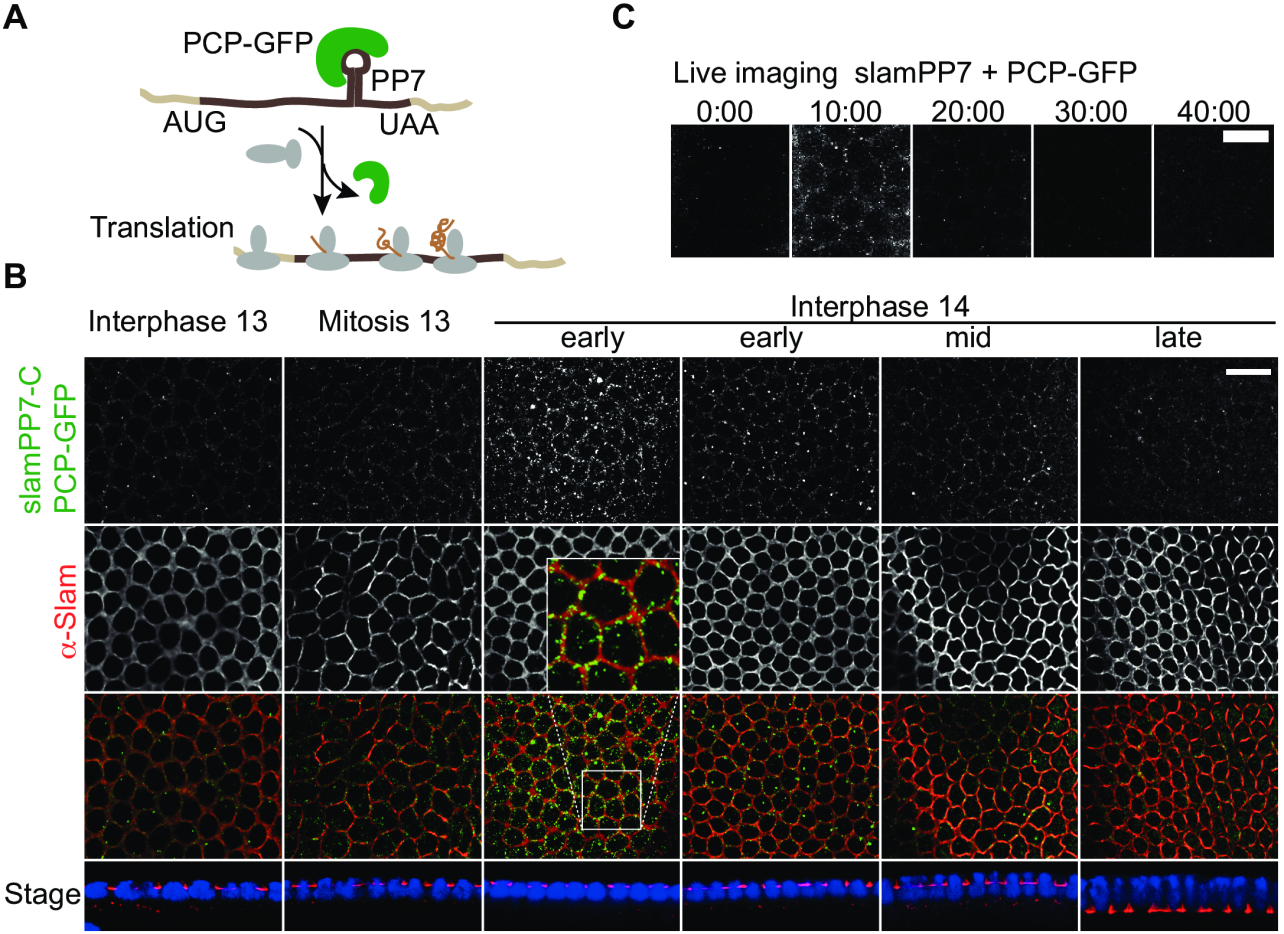
Dynamics of not yet translated *slam* mRNA. **(A)** Experimental scheme. Not yet translated *slam* mRNA is labeled by PCP-GFP binding to a PP7 site within the coding sequence prior to the first round of translation. During the first round of translation, PCP-GFP dissociates from *slamPP7* RNA. **(B)** Fixed embryos zygotically expressing *slamPP7* stained for PCP-GFP (green), Slam protein (red), and DNA (blue). Sagittal sections (Stage) allow staging. Scale bar = 10 μm. Inset at 2.25× magnification. **(C)** Images from time-lapse recording for PCP-GFP in embryos with zygotic expression of *slamPP7*. Time 0:00 min is approximately at mitosis 13. Scale bar = 5 μm.

Next, we addressed the spatial and temporal dimension of Slam protein accumulation at the basal domain. Protein accumulation may be due to recruitment of Slam protein from the cytoplasm to the basal compartment or, alternatively, to local translation of *slam* RNA. To distinguish these 2 options, we employed a recently developed method to fluorescently label not yet translated mRNA molecules [21, 22]. We generated genomic transgenes with a bacteriophage PP7 (PP7) hairpin loop inserted in the *slam* coding sequence (Fig 3A, S1 Fig). We inserted the PP7 sites close to the stop codon of the mRNA in order to assay all *slam* transcripts, including the mRNAs, which have initiated but not completed translation. Corresponding transcripts bind a bacteriophage PP7 coat protein (PCP)-GFP marker protein only until a ribosome has moved over the PP7 site [21, 22]. Thus, only not yet translated transcripts are labelled by PCP-GFP fluorescence. The *slamPP7* RNA shows a spatial and temporal expression pattern similar to endogenous *slam* RNA, persisting until the second half of cellularization (S2D Fig). In fixed embryos, we detected a dotted PCP-GFP signal (Fig 3B). PCP-GFP staining largely colocalized with Slam protein at the basal region. The staining was dynamic and only visible in embryos in early cellularization. Time-lapse imaging allowed a precise timing of PCP-GFP dynamics (Fig 3C). The dotted signal was largely observed during the onset of cellularization and quickly disappeared during the following minutes. These data show that a proportion of *slam* mRNA molecules reaches the basal domain before the first ribosomes have passed 3′-located PP7 sites and the first round of translation has been completed. Correspondingly, these data suggest that the synthesis of at least a fraction of the Slam protein molecules is completed at the FC. As the localized PCP signal quickly disappears, much fewer or no new and not yet fully translated *slam* RNA molecules reach the FC during cellularization.

### Slam protein is required for *slam* RNA localization

**Fig 4:**
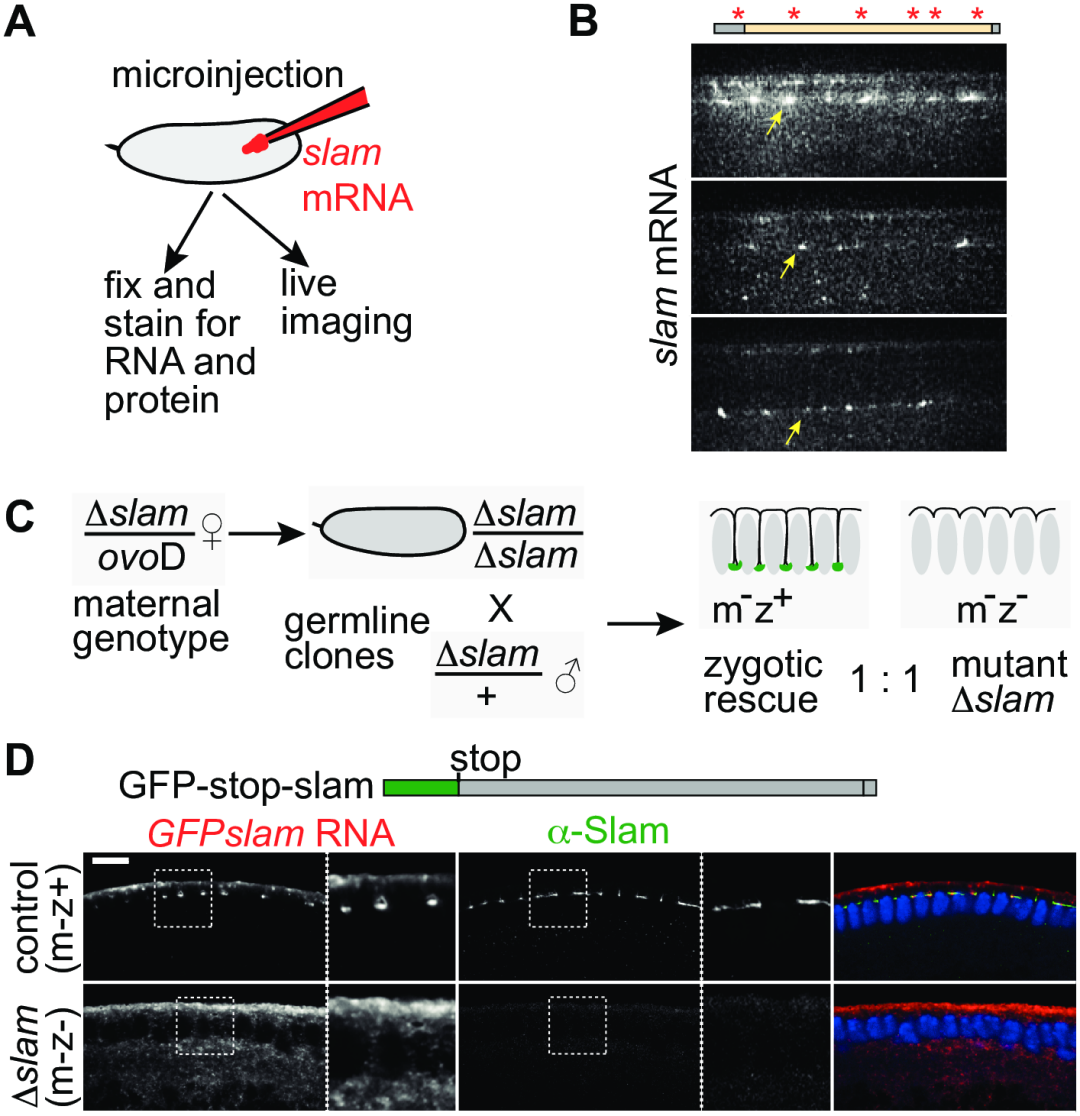
Slam protein is required for *slam* RNA localization. **(A)** Experimental scheme. Wild-type embryos were injected with synthetic *slam* mRNAs. After incubation, embryos were imaged or fixed and stained. **(B)** Fluorescently (schematically indicated by *) labeled *slam* RNA. Images from time-lapse recording. Arrows point to RNA at the FC. **(C)** Crossing scheme for generation of *slam* deficient (Δ*slam*) embryos. Half of the embryos receive the Δ*slam* allele and are maternally and zygotically deficient for *slam*; the other half receives a wild-type allele from the male and is zygotically rescued. **(D)** *GFP-stop-slam* RNA was injected into embryos from *slam* germ line clones. Fixed embryos were stained for the injected *GFP-stop-slam* RNA (grey/red), Slam protein (grey/green), and DNA (blue). Inset: areas marked by dashed box are shown at 2.25× magnification. Scale bars = 10 μm.

Given the peculiar temporal and spatial restrictions of *slam* translation, we functionally analyzed the role of RNA protein colocalization. We established a complementation assay either with injection of in vitro transcribed RNA or with zygotic expression from a transgene (Fig 4A). Injected *slam* RNA with a fluorescent label accumulated at the basal region as revealed by time-lapse imaging (Fig 4B, S1 Movie). Localization of the injected RNA and its derived protein was confirmed by staining for RNA and protein in fixed embryos. We did not observe an influence of GFP or myc tags on RNA and protein localization (S4A Fig). RNA and protein localization was also reconstituted in embryos lacking any endogenous *slam* RNA and protein (S4A Fig). *slam* deficient embryos were recognized by the absence of endogenous *slam* RNA and protein and staining at the injection site. These experiments demonstrate that the localization of *slam* RNA and colocalization of *slam* RNA and protein can be reconstituted in vivo.

In a simple model, *slam* RNA would accumulate independently of Slam protein at the basal region. Alternatively, Slam protein may be involved in the localization of its mRNA. To distinguish these 2 options, *slam* RNA with an early stop codon (GFP-stop-slam RNA) (Fig 4C) was introduced into embryos that are maternally and zygotically deficient for the *slam* locus (“m−z−”[17]). Such embryos were derived from females with *slam* germ line clones crossed to *slam* heterozygous males. Fifty percent of the embryos from such a cross are *slam* (”m−z−“) deficient and 50% of the embryos zygotically express *slam* (“m−z+,” zygotic rescue) (Fig 4C). These 2 genotypes were easily distinguished by staining for endogenous Slam protein and by their morphology. The zygotically rescued embryos served as an internal reference for the injection and staining procedure. We could score for localization of the injected RNA also in *slam* deficient embryos, because the FC/basal domain is specified even in the absence of *slam* [17]. We did not detect localizing RNA in *slam* deficient embryos. The signal for GFP-stop-slam RNA was uniformly distributed at the apical surface. In contrast, in control embryos, the injected RNA colocalized with endogenous Slam protein (Fig 4D). The GFP-stop-slam RNA localization in control embryos may be due to an interaction with Slam protein or with the endogenous *slam* RNA. We favor the first option, because injected *slam* RNA encoding functional Slam protein is sufficient for *slam* RNA and protein localization in embryos lacking endogenous *slam* RNA (S4A Fig). Taken together, these experiments show that Slam protein is required for *slam* RNA localization at the FC.

### Slam protein has an intrinsic RNA-independent affinity for the FC

**Fig 5:**
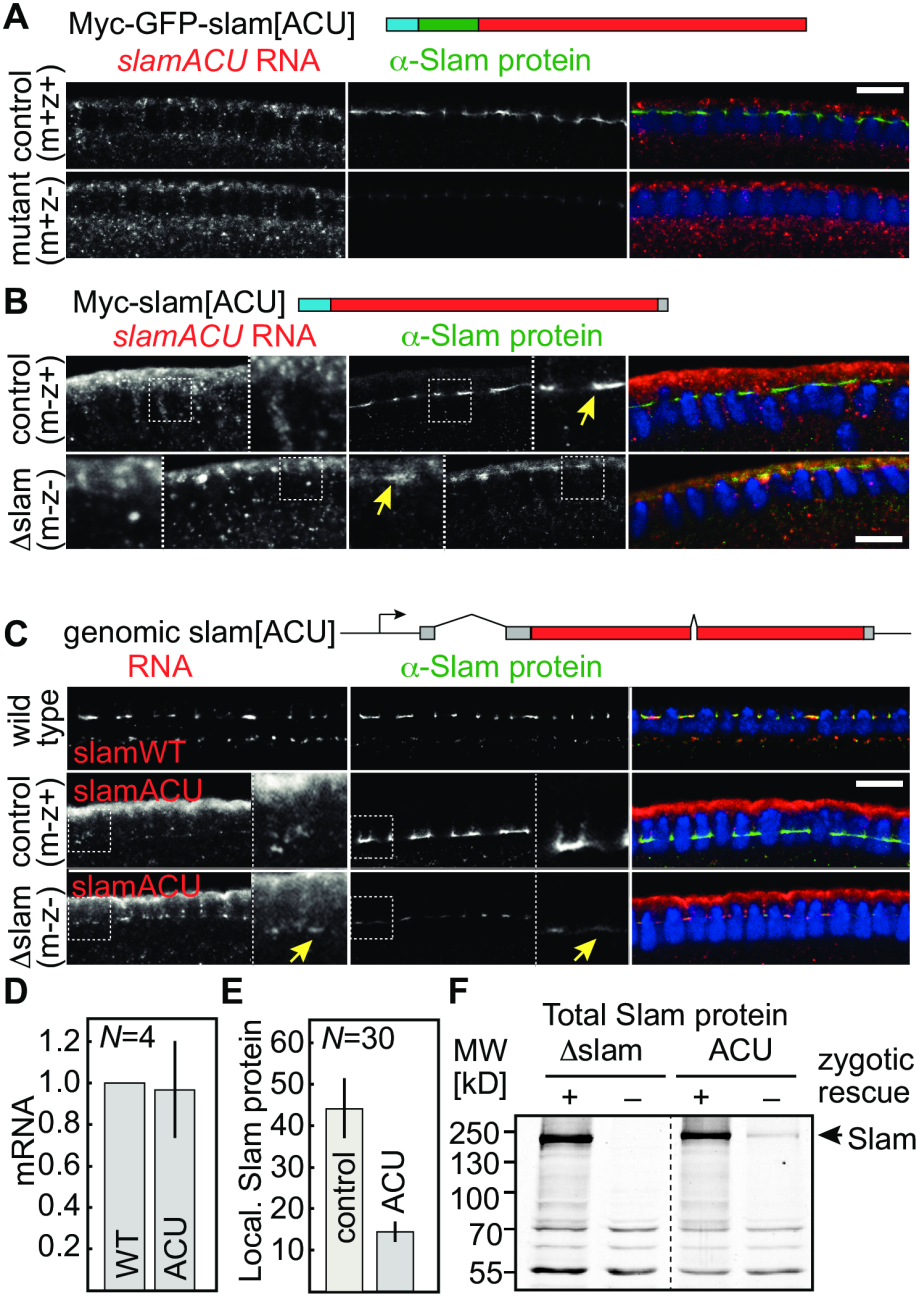
RNA localization and expression of *slam* with alternative coding. **(A—C)** Fixed embryos stained for *slam* or *slam*[ACU] RNA (grey/red), Slam protein (grey/green), and DNA (blue), as indicated. **(A)** Myc-GFP-*slam*[ACU] was expressed in zygotically rescued and *slam* deficient embryos from a transgene driven by maternal Gal4. **(B)** Zygotically rescued and *slam* deficient embryos from *slam* germ line clones injected with myc-*slam*[ACU] RNA. Arrows point to Slam protein localization at the FC. **(C, E)** *slam*[ACU] genomic transgene was expressed in zygotically rescued (m−z+) and *slam* deficient embryos (m−z−) from *slam* germ line clones. **(C)** Arrows point to *slam*[ACU] RNA and Slam protein localizing at the FC. WT embryos were stained for endogenous *slam* RNA and protein in a separate tube. **(D)** Quantification of *slam* WT (endogenous gene) and ACU RNA (from transgene) abundance by reverse transcription and qPCR within the same embryos (2 copies of *slam*[WT] and 2 transgenic copies of *slam*[ACU]. Expression of *slam*[ACU] was normalized to *slam*[WT]. **(E)** Quantification of fluorescence staining for Slam protein at the FC for images shown in panel C. Control (m−z+), ACU (m−z−with ACU transgene). Three embryos with 10 furrows each, student *t* test *P* = 3.5 × 10^−29^. Arbitrary units. **(F)** Western blot of extracts from manually staged and genotyped embryos from *slam* germ line clones with or without expression of *slam*[ACU] from the genomic transgene. Zygotically rescued embryos express Slam from the endogenous gene. Scale bars = 10 μm. Error bars indicate standard deviation. The underlying data for this figure can be found in S1 Data. kD, kilodalton; MW, molecular weight; *N*, number of biological replicates; qPCR, quantitative polymerase chain reaction.

Next, we asked whether *slam* RNA localization is required for protein localization. For this, we mapped multiple regions within the 5′ untranslated region and the coding sequence, which are sufficient for localization at the FC (S4 Fig). Given the complexity of multiple parts contributing to RNA localization, we generated a novel *slam* gene, *slam* alternative codon usage ([ACU]), in which a majority of the codons were replaced by synonymous codons (Fig 5A, S1 and S5 Figs). *slam*[ACU] RNA lost the ability to localize to the FC (Fig 5A and 5B). *slam*[ACU] RNA was uniformly distributed in the cytoplasm in the presence or absence of endogenous *slam*, when expressed from a transgene (myc-GFP-slam[ACU]) or injected as synthetic RNA (myc-*slam*[ACU]) (Fig 5A and 5B). These data indicate that our mutagenesis strategy successfully impaired the FC localization of *slam* RNA.

Having generated a nonlocalizing *slam[ACU]* RNA, we could test whether Slam protein accumulation at the FC would depend on RNA localization. We stained embryos injected with the nonlocalizing *slam*[ACU] mRNA for Slam protein (Fig 5B). *slam* embryos were recognized by the absence of overall *slam* RNA or protein signal. Staining restricted to the injected site is due to the injected construct. We clearly detected Slam protein at the FC in *slam* embryos (Fig 5B). We conclude that Slam protein has an intrinsic RNA-independent affinity for the FC. These data also indicate that we preserved the coding capacity of *slam*[ACU].

### Translational control of *slam* at the FC

We got the impression that protein levels with *slam*[ACU] were lower than with wild-type *slam*. The difference may be due to inefficient translation of *slam*[ACU], to inefficient localization of Slam protein, or to the injection procedure resulting in lower mRNA levels than endogenous *slam* expression.

In order to test translation efficiency in embryos, we generated a genomic transgene with the *slam*[ACU] sequence, preserving introns, 5′ untranslated regions, and 3′ untranslated regions (Fig 5C, S1 Fig). A corresponding genomic transgene with the endogenous coding sequence fully complements a *slam* deficiency [17]. *slam*[ACU] RNA expressed from the genomic transgene was comparably abundant as the endogenous wild-type allele (Fig 5D), confirming the integrity of the transgene and transcript. *slam*[ACU] RNA showed a low degree of FC localization in wild-type background and slightly more so in *slam* deficient embryos (Fig 5C). This low degree of localization is likely due to the 5′ untranslated region, which is sufficient to localize at the FC in the injection assay (S4B Fig).

Next, we assayed protein levels by embryo staining and western blot with total extracts. Staining for Slam protein in *slam*[ACU] embryos in comparison to rescued siblings revealed strongly reduced protein levels at the FC (Fig 5C and 5E). A similarly clear difference in total protein levels was detected by western blot (Fig 5F). For this, we manually sorted embryos according to stage (mid-cellularization) and genotype. No Slam was detected in *slam* deficient embryos (Fig 5F). *slam*[ACU] embryos contained much less Slam protein than the rescued siblings, which zygotically expressed Slam from the endogenous gene (Fig 5F). These data indicate Slam protein derived from *slam*[ACU] is much less abundant and that *slam*[ACU] was thus much less translated than wild-type *slam*.

The reduced translation may be due to secondary RNA structures or codon usage affecting translation efficiency. We distinguished these options by expression of *slam*[ACU] in comparison to *slam*[WT] in cultured *Drosophila melanogaster* Schneider 2 cells (S2 cells). S2 cells do not express Slam protein in detectable levels (S6A Fig). Following transient transfection, we detected comparable *slam* RNA and protein levels for *slam*[ACU] and wild-type *slam* (S6B and S6C Fig). These data indicate that generic translation in S2 cells is comparably efficient for *slam*[ACU] and wild-type *slam*. These data do not rule out the conceivable option that *slam*[ACU] contains secondary RNA structures or peculiar codon usage affecting translation efficiency that are specifically present in the embryo but not in S2 cells.

Consistent with the reduced protein expression from *slam*[ACU] in embryos, the *slam*[ACU] genomic transgene only partially complemented the cellularization phenotype and did not rescue the lethality of a *slam* deficiency. Embryos maternally and zygotically deficient for *slam* but with *slam*[ACU] only formed a short cellularization furrow and did not complete cellularization (S7 Fig). This functional test showed that efficient expression of *slam* is physiologically important. Taken together, these data suggest that localization of *slam* RNA at the basal domain or its colocalization with Slam protein are important for full translation.

### Slam protein attracts *slam* mRNA to the FC

**Fig 6:**
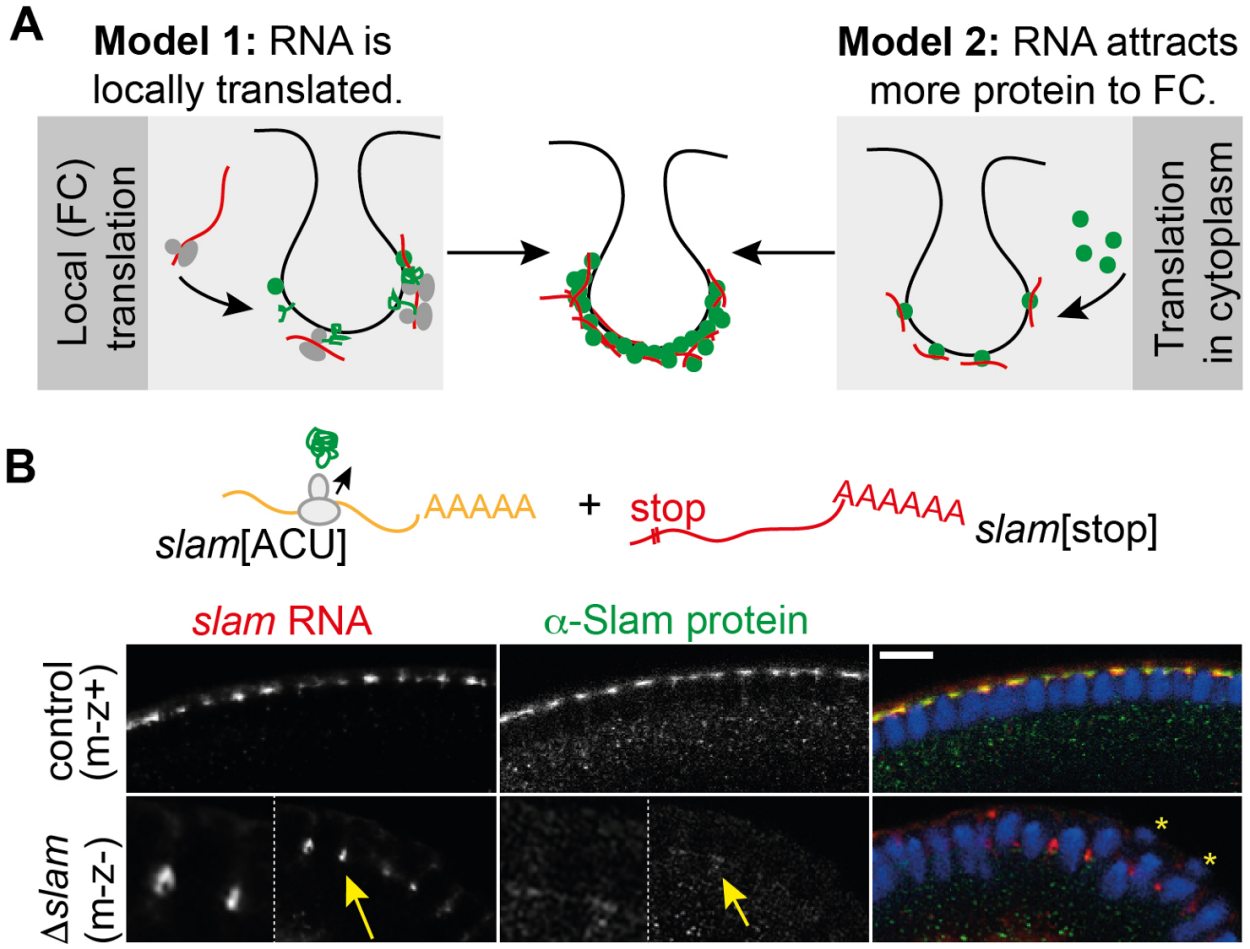
Slam protein attracts *slam* RNA to the FC. **(A)** Two models for accumulation of *slam* RNA and protein. RNA (red) with ribosomes, protein (green dots). Protein is synthetized by local translation after the RNA was attracted by protein (Model 1). Protein is synthesized in the cytoplasm and attracted by localized RNA (Model 2). **(B)** Test of these models by a nontranslatable, localizing RNA (red) and a translatable, localization-impaired RNA (orange). Embryos from *slam* germ line clones with genomic *slam*[ACU] injected with GFP-stop-*slam* RNA and stained for the injected RNA (grey/red), Slam protein (grey/green), and DNA (blue). Insets are at 2.25× magnification. Yellow arrows point to localized RNA and protein. * marks posterior pole cells. Scale bars = 10 μm.

The transient FC accumulation of not yet translated *slam* RNA suggests a local translation of *slam*, at least partially (Model 1, Fig 6A). In addition to local translation, *slam* RNA may recruit Slam protein to the basal region, which was synthesized by translation within the cytoplasm (Model 2, Fig 6A). To functionally assess the significance of Model 2, we injected a translation-incompetent but localization-competent *slam* RNA (GFP-stop-slam) into the *slam*[ACU] embryos (Fig 6B). We would expect localization of the injected RNA and in the case of Model 2, a corresponding local increase of Slam protein levels.

We detected the injected GFP-stop-slam RNA at the FC in levels comparable to *slam* in control embryos (Fig 6B). Slam protein staining was uniform in low levels, similar to *slam*[ACU] embryos (Fig 6B). We did not detect any increased signal at the injection site. These data suggest that localized *slam* RNA does not attract cytoplasmic Slam protein to the FC.

### Biochemical association of *slam* mRNA and protein

**Fig 7:**
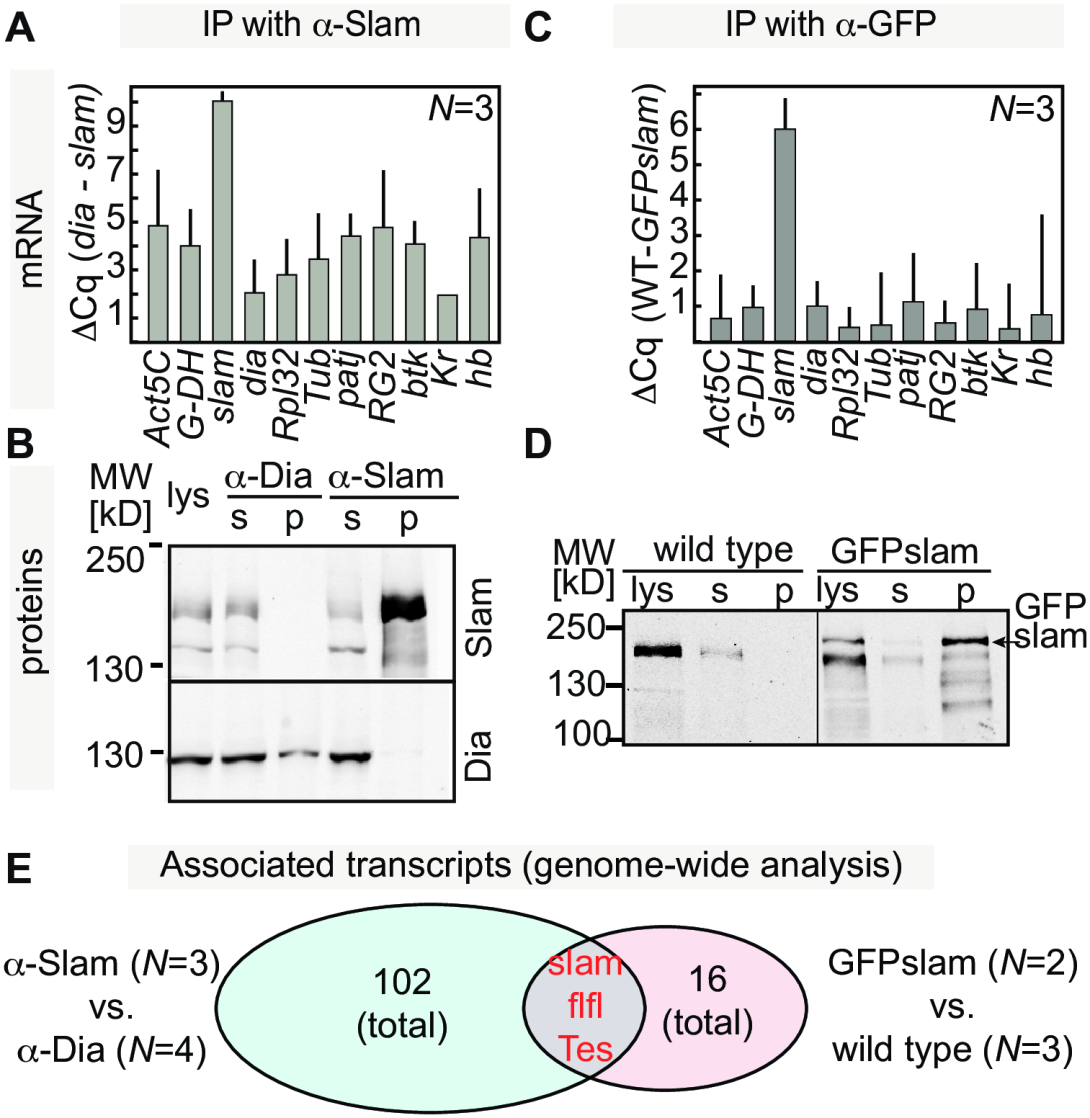
Biochemical association of *slam* RNA and protein. Immunoprecipitation from **(A, B)** wild-type extracts with Slam and Dia antibodies or from **(C, D)** GFP-slam and wild-type extracts with GFP antibody. **(A, C)** Associated RNA of selected genes were quantified by RT-qPCR. Error bars indicate standard error of mean. The underlying data for this figure can be found in S1 Data. **(B, D)** Slam and GFP-slam proteins were detected by western blot with Slam antibody. **(E)** Transcripts associated with immunoprecipitates were identified by next generation sequencing. An enrichment factor was calculated for each transcript as the ratio of transcript numbers between immunoprecipitates (1) with Slam and Dia antibody and wild-type lysates or (2) with GFP binder and lysates from GFP-slam and wild-type embryos. Indicated are the number of transcripts above threshold (4-fold enrichment). The intersection contains 3 transcripts. ΔCq, difference in qPCR cycles; GFP, green fluorescent protein; *N*, number of biological replicates; RT-qPCR, reverse transcription quantitative polymerase chain reaction.

A key feature of *slam* is, on the one side, the functional interdependence of RNA and encoded protein and, on the other side, the temporally and spatially restricted translation. We hypothesized that the functional interaction of *slam* RNA and protein is based on a biochemical interaction. By Slam immunoprecipitation, we found that *slam* mRNA was enriched in the bound fraction as compared to immunoprecipitates by Dia antibodies (Fig 7A and 7B). The formin Dia nucleates and elongates F-actin and is enriched at the FC [23–25]. The enrichment of *slam* RNA was higher than for a series of control RNAs (Fig 7A). We confirmed the specific association of *slam* RNA with Slam protein by conducting immunoprecipitation with single-chain GFP antibody (GFP-binder) and lysates of embryos expressing GFP-slam from a genomic transgene in comparison to wild-type extracts (Fig 7C and 7D). We found a higher enrichment of *slam* mRNA than other RNAs in the bound fraction (Fig 7C). This enrichment confirmed the specificity of the RNA-protein interaction.

To assess whether the bound fraction contained other mRNAs beside *slam* RNA, we identified associated transcripts in an unbiased manner by next generation sequencing (Fig 7E). RNA was isolated and sequenced from the bound fractions of the immunoprecipitation experiments with Slam antibody and GFP-binder, including the controls. In the bound fractions, we detected 102 and 16 transcripts, respectively, which were enriched more than 4-fold (Fig 7E, S1 Table). The intersection of the 2 experimental approaches contained *slam* with the strongest enrichment and 2 more unrelated transcripts. These data indicate that *slam* mRNA is present in a specific complex with Slam protein in embryos. Although *slam* is among the most abundant transcripts during this stage, none of the other abundant transcripts were enriched in our immunoprecipitation experiments (S1 Table). As the primary structure of Slam does not contain any obvious structural domains related to RNA binding, other RNA binding proteins likely mediate the biochemical association of *slam* RNA and protein.

## Discussion

The primary function of *slam* RNA is encoding Slam protein. In addition to coding information, *slam* RNA contains 2 more pieces of noncoding information: (1) information for specific subcellular localization of the RNA at the FC, which is at least partially mediated by an interaction with Slam protein, and (2) information for spatial and temporal control of translation, which is high at the FC during the onset of cellularization. By comparing wild-type RNA with a variant RNA, *slam*[ACU] with the same coding information, the relevance of the noncoding information was uncovered. *slam*[ACU] RNA is widely distributed in the cell and gives rise to much less Slam protein. Containing coding and noncoding information distinguishes *slam* RNA from generic mRNAs, which contain only coding information. *slam* RNA may be related to the growing class of mRNAs with coding and noncoding functions (cncRNA) [26].

Using the injection assay with synthetic RNA transcribed from truncation constructs, we identified several regions of *slam* RNA that are sufficient for localization to the FC in wild-type embryos. This includes the 5′ untranslated region and at least 2 large regions within the coding sequence, which we have so far not further defined. Each of these regions is able to localize to the FC on its own in wild-type embryos, which indicates redundancy in the mechanism of RNA localization. Interpretation of these data is difficult, however, given that endogenous *slam* RNA and protein were present in our assay, which may lead to localization by oligomerization or other indirect binding mechanisms.

*slam* RNA is subject to spatiotemporal control of translation. Although the RNA is present in high levels during the first half of cellularization, translation is restricted to the initial minutes of cellularization. The almost constant protein levels throughout cellularization are due to the stability of Slam protein, as inhibition of new synthesis by cycloheximide leads only to a weak decrease of GFP-slam fluorescence. In contrast to these constant levels during cellularization is the sharp increase in protein levels during the onset of cellularization.

This initial rise in protein levels is partly due to the transcriptional up-regulation of *slam*. The transcriptional regulation appears not to be sufficient, as we observed a striking difference between *slam*[wild-type] RNA with *slam*[ACU] RNA. Although both RNAs contain the same coding information and give rise to similar amounts of Slam protein in cultured cells, *slam*[wild-type] RNA is more efficiently translated than *slam*[ACU] RNA in blastoderm embryos. Based on the correlation of impaired RNA localization and reduced translational efficiency, we favor the model that efficient translation is linked to RNA localization or interaction with Slam protein at the FC. However, the embryo-specific lower efficiency of *slam*[ACU] translation may alternatively be due to secondary RNA structures or disadvantageous codons, which were introduced in our mutagenesis.

A particular feature of *slam* is that the encoded protein is required for the elaboration of noncoding features. *slam* RNA requires Slam protein for accumulation at the FC. Not only do we observe a functional interaction of the RNA and protein but also colocalization and biochemical association, indicating molecular interactions. These molecular interactions between RNA and protein may be direct or indirect. They are likely to be indirect, as Slam protein does not contain a canonical RNA binding domain or does not share any detectable sequence homology to RNA binding proteins. Analysis of transcripts associated with Slam protein provided distinct lists depending on the experimental procedure. Importantly, *slam* RNA was identified by both approaches, which is consistent with our quantitative polymerase chain reaction (qPCR) analysis for a few selected genes. The list of associated transcripts contained transcripts with high and low abundance, indicating that the procedure was sufficiently sensitive to also detect weakly expressed genes. Screening through the gene functions, we did not detect a specific set of genes, such as genes involved in cellularization or cytoskeletal organization.

**Fig 8:**
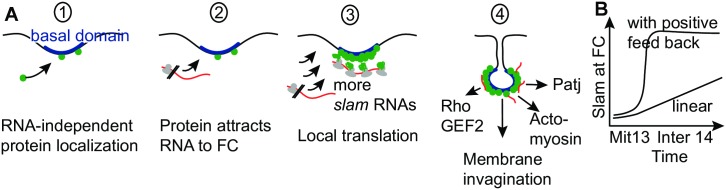
Model for spatiotemporal dynamics of *slam* RNA and protein. **(A)** Conceptual steps for accumulation of *slam* RNA and protein at the basal compartment/prospective FC. **(B)** Scheme for time course of Slam accumulation at the FC with a positive feedback or according to a linear model. Mit13, mitosis of cycle13; Inter 14, interphase of cycle 14.

The biological function of the intimate relationship of *slam* mRNA and its protein has been unclear. Given the observed specific spatiotemporal profile of *slam* translation and Slam dynamics, we propose the following model (Fig 8A). Initially, Slam protein accumulates in low levels at the FC independently of its mRNA. Starting with the onset in zygotic transcription, *slam* mRNA exits the nucleus as part of a complex that is not competent for efficient translation. At least a fraction of *slam* RNA molecules accumulates at the basal domain forming the FC prior to the first round of translation. At the basal domain, *slam* mRNA becomes competent for efficient translation, which leads to an increase in Slam protein at the FC. The increased amounts of Slam protein further promote accumulation of *slam* mRNA, leading to even more Slam protein. Such a mechanism constitutes a positive feedback loop, which provides an explanation for the observed switch-like profile of Slam protein staining at the FC (Fig 8B). Some minutes later, when full Slam levels have been reached, translation is turned off. Slam protein then functions in spatially restricted activation of Rho signaling, actomyosin organization, Patj recruitment, cortical compartmentalization, and furrow invagination (Fig 8) [10, 17, 25]. As *slam* is a key regulator of cellularization, the rapid increase of Slam protein may be important for a coordinated and timely onset of its downstream processes.

We identified *slam* as an mRNA with noncoding information for localization and translational control in addition to its coding information. On the molecular level, *slam* is special in that mRNA and protein associate in a complex as demonstrated by co-immunoprecipitation. This unconventional relationship of *slam* RNA and protein may be important for the tightly restricted subcellular localization and strong increase in protein levels at the FC. The functional interaction of *slam* RNA and protein constitutes a positive feedback loop, which contributes to the fast increase in Slam protein levels at the FC.

## Materials and methods

### Genetics

Fly stocks were obtained from the Bloomington stock center, if not otherwise noted. The following fly strains and mutations were used: Df(2L)slam Frt^2L^ slam5′rescue [17], *nuf*^1^ [18], *shi* [20]. The following transgenes were used: *slam*[wild-type], genomic transgene with the *slam* locus [17], GFP-slam, genomic transgene with GFP at N-terminus, *slam*[ACU], genomic transgene with alternative coding sequence, *slamPP7*, genomic transgene with PP7 sites inserted within the coding sequence, UASp-myc-GFP-slam[wild-type], UASp-myc-GFP-slam[ACU], transgenes with GAL4 driven expression. Genomic transgenes were generated by PhiC31 integrase-mediated site-specific insertions on the third chromosome at cytological position 68A4 [27]. *slam* deficient embryos were generated by *slam* germ line clones in progenies of the cross Df(2L)slam Frt^2L^ slam5′rescue/CyO females with hs-Flp^122^; *ovo*^D2L^ Frt^2L^/CyO males and heat shock for 2× 1 h at 37°C in larvae [17]. The Df(2L)slam Frt^2L^ slam 5′rescue is also deficient for some proximal genes in addition to the *slam* locus. These genes do not function in early embryonic development, as the cellularization phenotype is rescued with the genomic *slam* transgene [17]. For microinjection experiments, females with *slam* germ line clones were crossed with males Df(2L)slam Frt^2L^ slam5′rescue/ CyO. In the case of transgenes, females with *slam* germ line clones (hsFlp^122^/+; Df(2L)slam Frt^2L^ slam5′rescue/ovo[D2L] Frt_2L_; transgene/+ were crossed with males Df(2L)slam Frt^2L^ slam5′rescue/CyO; transgene/transgene. Rescue of viability was tested with Df(2L)slam Frt^2L^ slam5′rescue/Df(2L)slam Frt^2L^ slam3′rescue; transgene/+ [17]. All embryos from crosses with *slam* germ line clones are maternally deficient for *slam*. 50% of the progeny are also zygotically deficient for *slam*, whereas the other half contain a zygotic wild-type allele of *slam* (zygotic rescue). Embryos with zygotic rescue form a furrow during cellularization and have strong and uniform *slam* RNA and protein expression. For imaging of not yet translated *slam* RNA, embryos were obtained from females expressing PCP-GFP [21, 22] crossed to males with the *slamPP7* transgene. Mapping of the RNA localization regions was performed with wild-type flies (OregonR). The *shibire* phenotype was induced in embryos from heterozygous females by shifting the embryos to 32°C 10 min prior to the FRAP experiment [17].

### Molecular genetics

Truncations and gene fusions of *slam* cDNA as specified in Table 1 were cloned by PCR-based cloning. The vector pCS2 or pCS2-6xmyc (obtained from R. Rupp) contains a short leader sequence derived from the beta globin leader sequence. Genomic constructs were cloned into a pattB plasmid, suitable for generation of transgenic flies [27]. Details of the cloning procedures and PCR are available on request. cDNA constructs were based on cDNA clone LD22808, which served as template for PCR. In comparison to other cDNA clones available now, LD22808 lacks 69 nucleotides at position CDS 1083, which do most likely represent an additional, rarely used intron. The *slam* gene with alternative codon usage (*slam*[ACU]) was designed by each 3-nucleotide codon with another suitable codon, according to the codon usage frequency in *Drosophila* [28] and synthesized by MWG Eurofins. Noncoding parts of the gene (5′ and 3′ untranslated regions and introns) were not mutated. *slam*[ACU] was cloned into the pattB-slam8.6 genomic construct [17] by PasI and SphI restriction sites. Introns were preserved. For *slamPP7*, a sequence with 12xPP7 [21, 22] was inserted at the unique SacII site of the coding sequence within the 8.6-kb genomic DNA of the *slam* locus cloned in pBKS and transferred to the transformation vector pattB. This construct also contained a 6x MS2 site inserted at position 50 of the 3′ untranslated region. For the transgenes with GAL4 driven expression pUASp-myc-GFP-slam and pUASp-myc-GFP-slamACU, *slam* and *slam*[ACU], cDNAs were cloned in frame into pUASp-myc-GFP.

**Table 1:** List of plasmids.

Name	Type	Description (nt of CDS)
CS-slam	Plasmid/RNA	Full length cDNA* (LD22808)
Slam-5	Plasmid/RNA	slam 5′UTR-(1..3,519)-Myc
Slam-6	Plasmid/RNA	Myc-slam(1..3,522)-3’UTR
Slam-8	Plasmid/RNA	Myc-slam(1..3,519)-myc
Slam-5′UTR-GFP	Plasmid/RNA	5′UTR-GFP
Slam-1	Plasmid/RNA	5′UTR-slam(1..904)-myc
Slam-2	Plasmid/RNA	Myc-slam(598..1,884)
Slam-3	Plasmid/RNA	Myc-slam(1,521..2,826)
Slam-4	Plasmid/RNA	Myc-slam(2,431..3,522)-3′UTR
Slam-19	Plasmid/RNA	GFP-slam(1..3,519)-myc
Slam-14	Plasmid/RNA	GFP-TGA-slam(1..3,519)-myc
Slam-16	Plasmid/RNA	GFP-slam(598..1,884)
Slam-20	Plasmid/RNA	GFP-slam(598..990)
Slam-21	Plasmid/RNA	GFP-slam(991..1,428)
Slam-17	Plasmid/RNA	GFP-slam(1,498..1,884)
Slam-22	Plasmid/RNA	GFP-slam(1..597)
Slam-26	Plasmid/RNA	GFP-slam(1..597+1,885..3,522)-myc
SlamΔBam	Plasmid/RNA	GFP-slam(1..507+1,576..3,522)-myc
Slam-24	Plasmid/RNA	GFP-slam(2,433..3,522)-3′UTR
Slam8.6	Genomic transgene	Wild-type sequence of *slam* locus, 8.645 nt
Genomic GFP-slam	Genomic transgene	Wild-type sequence of *slam* locus. GFP and a TEV cleavage site are inserted at start codon.
SlamACU	Genomic transgene	Complete CDS is substituted by ACU sequence, 5′UTR, 3′UTR, introns and the 69 nts at CDS1083 are similar to wild-type slam8.6.
SlamPP7	Genomic transgene	Wild-type sequence of *slam* locus. A sequence with 12x PP7 was inserted at position 3,460.
CS-slamACU	Plasmid/RNA	5′UTR-slamACU(1..3,519)-myc
CS-myc-slam-ACU	Plasmid/RNA	Myc-slamACU(1..3,522)
UASp-myc-GFP-slam	Transgene	*Slam* cDNA fused in frame to 6xmyc and GFP.
UASp-myc-GFP-slamACU	Transgene	*SlamACU* cDNA fused in frame to 6xmyc and GFP.
CS-GFP	Plasmid/RNA	GFP

*All cDNA constructs are based on cDNA clone LD22808, which lacks 69 nucleotides at position CDS1083/1084. These 69 nucleotides are included in other cDNA clones and in the genomic constructs and most likely do not represent an additional intron.

**Abbreviation:** ACU, alternative codon usage; CDS, coding DNA sequence; CS, vector plasmid CS2; GFP, green fluorescent protein; nt, nucleotide; PP7, bacteriophage PP7; *slam*, *slow as molasses*; TEV, Tobacco Etch Virus nuclear-inclusion-a endopeptidase; UAS, Upstream Activation Sequence; UTR, untranslated region

### RNA synthesis in vitro

For microinjection, capped transcripts were synthesized with linearized plasmid templates and the SP6 mMESSAGE mMACHINE high yield capped RNA transcription kit (Applied Biosystems). For live imaging of injected RNA, the reaction mix was complemented with 0.5 μl aminoallyl-UTP (25 mM, Jena bioscience). Isolated RNA (4 μl) was labeled with 5 μl Rhodamine Red-X, (succinimidyl ester in 56 μl DMSO, Invitrogen), 1 μl 0.1 M Na-Borate [pH 9] at RT overnight. The labeling dye and salts were removed using a desalting Sephadex G50 spin column. For RNA in situ hybridization, *slam* RNA probes were generated with T7 RNA polymerase and antisense templates as previously described [10].

### RNA extraction, reverse transcription, quantitative PCR, RNA sequencing

Total RNA was extracted from staged embryos using Trizol Reagent (Invitrogen). Reverse transcription was performed with 1–2 μg RNA and oligo-dT primers according to the instructions of the manufacturer (Roche). Two μl (12.5% of the sample) of reverse transcripts were analyzed by quantitative PCR with specific primers. qPCR reactions were performed in duplicates. Specificity was controlled by a sample in which reverse transcriptase enzyme was omitted and in reactions with defined amounts of templates. The following primer pairs were used for quantitative PCR: WT1 SY88 (603–622) + SY89 (885–903), WT2 SY94 (−334–−316) + SY95 (−24–−1), ACU SY92 (603–620) + SY93 (886–905). ACU is specific for the *slam*[ACU] allele. The numbers in parentheses specify the position of the nucleotide within the cDNA, according to the coding sequence. For genome-wide analysis, RNA extracted from immunoprecipitates was subjected to next generation sequencing (Illumina HiSeq2000), according to the manufacturer’s protocols. Analysis was performed with 2–4 biological replicates. Weakly expressed genes (*N* < 50 or *N* < 10 for the experiment with Slam/Dia antibodies or GFP binder, respectively) were not considered in the analysis. RNA expression data were obtained from wild-type embryos with 1.5–2.5 h ([29], GEO Series accession number GSE97557). The data from Next Generation Sequencing have been deposited in NCBI’s Gene Expression Omnibus [30] and are accessible through GEO Series accession number GSE99761 (https://www.ncbi.nlm.nih.gov/geo/query/acc.cgi?acc=GSE99761.

### Histology

Embryos were fixed in 8% formaldehyde in phosphate-buffered saline (PBS) and fluorescent RNA in situ hybridization was performed as previously described [10]. Protein staining with a specific antibody was performed after RNA staining. The following antibodies were used: primary antibodies: rabbit/guinea pig-α-Slam [10], mouse-α-myc-9E10 (Roche); secondary antibodies: Alexa-coupled goat-anti-mouse/rabbit/guinea pig antibodies (Invitrogen), peroxidase coupled α-digoxigenin Fab-fragments (Roche). RNA probes against *slam* cDNA sequence, against *slam*[ACU], against *GFP*, or against *PP7* were used. DNA was costained by DAPI. Embryos were mounted in Aquapolymount.

### Microscopy

Fluorescent images of fixed and immune-stained embryos and live imaging experiments were recorded with a confocal microscope (Zeiss LSM780). Images were processed with ImageJ/Fiji and Photoshop (Adobe). Images from FRAP experiments were analyzed as previously described [17]. Time-lapse recordings of embryos injected with fluorescently labeled *slam* RNA were recorded with a Leica epifluorescence microscope. Time-lapse recordings with differential interference contrast optics were recorded with an inverted microscope (Zeiss Observer Z.1, 25× NA0.7/oil) with a computer-controlled stage, allowing simultaneous recordings of multiple embryos.

### Microinjections

Embryos were injected as previously described [10]. The mRNA constructs were injected at concentrations of about 2–5 μg/μl. Fixation was performed about 1–1.5 h after injection, when the majority of embryos were in the cellularization stage. Cycloheximide (1 mg/ml) or buffer (0.1 M Na-phosphate, 5 mM KCl) was injected from the posterior end. About 10 minutes later imaging or FRAP was started. The embryos contained a genomic GFP-slam transgene.

### Expression of *slam* and *slam*ACU in *Drosophila* Schneider cells

*slam* wild type and *slam*[ACU] (on pCS2 plasmid, driven by a CMV promoter) were tested for protein production levels in cultured *D*. *melanogaster* Schneider line 2 (S2) cells. S2 cells were grown in Schneider’s Drosophila medium (Gibco/Invitrogen) supplemented with 10% fetal bovine serum (Gibco/Invitrogen) at 25°C. Cells were reseeded 1 day before transfection at about 1.0 million cells/ml (counted in Neubauer chamber). Transfection was performed according to the instruction of the manufacturer (Qiagen Transfection Kit containing Effectene Transfection Reagent). A control reaction (using pCS-GFP) indicated an approximately 6% transformation efficiency. Cells were harvested after 48–72 h. Half of the cells were dissolved in SDS loading buffer (Laemmli) to a final concentration of 50,000 embryos/μl. SDS gel electrophoresis was performed with 1 million cells per sample lane. RNA was extracted from the second half of the cells using Trizol Reagent (Invitrogen).

### Co-immunoprecipitation

Guinea pig-α-Slam antibody [10] or guinea pig-α-Dia antibody [24] was bound to Dynabeads coated with Protein A (Invitrogen) in PBT for 1 h at 4°C. Beads were washed 5 times with PBT. Embryonic extracts were prepared by lyzing about 100 mg of 1.5–3-hour-old embryos in 1 ml YSS buffer [31] (50 mM Tris/HCl [pH8], 75 mM NaCl_2_, 1 mM MgCl_2_, 0.05% NP40, 100 mM sucrose, 1 M DTT, protease inhibitors [Roche], RNAase inhibitors) in a Dounce homogenizer. The lysate was centrifuged for 15 min at 14,000 rpm to remove debris. The cleared supernatant was mixed with the beads coated with antibody and incubated on a rotator for 1 h at 4°C. A 200-μl sample was taken from the supernatant (unbound fraction). Beads were then washed 4 times with cold YSS buffer. 200 μl of YSS buffer was added to the bound samples. Unbound and bound samples were complemented with 20 μl 10% SDS and 0.5 μl glycogen (20 μg/ml). Samples were extracted with phenol/chloroform/isoamylalcohol. Protein was precipitated from the organic phase with 1 ml acetone. RNA was precipitated by the addition of 40 μl Na-acetat (3 M) and 500 μl 100% ethanol to the aqueous phase. Protein and RNA samples were analyzed by western blot, quantitative RT-PCR, and next generation sequencing, respectively. For co-immunoprecipitation with the single-chain GFP antibody (GFP binder or GFP-TRAP, Chromotek), staged embryos from GFP-slam transgenic or wild-type flies were collected and lysed as described above, except for the following: 0.5 μl biotinylated-GFP binder (50 mM) was mixed with cleared embryo lysate for 1 h at 4°C. Then, 20 μl PBS-washed streptavidin-coupled Dynabeads were added and mixed for a further 1 h at 4°C.

### Western blots and analysis of manually selected embryos

SDS polyacrylamide gel electrophoresis (PAGE) and western blot were performed as previously described [10]. Primary antibodies were guinea pig or rabbit-α-Slam [10], mouse-α-tubulin-α (Sigma), and rabbit or guinea pig-α-Dia [22]. Western blots were developed with fluorescently labeled secondary antibodies (800CW Donkey-α-guinea pig/mouse/rabbit IgG) and detected with a LICOR system. Sixteen-bit images were processed by Photoshop and FIJI/ImageJ. For analysis of embryos with defined genotype and stage, embryos were heat fixed as previously described [10], stained for Slam and DAPI, and mounted in 50% glycerol. Under fluorescent microscope, embryos in cellularization were sorted by their zygotic genotype and stage. Sorted embryos (*N* = 10–20) were lysed in Laemmli buffer with a Dounce homogenizer and analyzed by western blot.

## Supporting information

S1 Fig *slam* constructs and transgenes. Schematic drawing of genomic *slam* transgenes and *slam* cDNA constructs in comparison to the *slam* locus. Boxes indicate transcribed regions. Coding sequence is marked in light orange, sequences with alternative coding in red, untranslated region in grey, GFP in green, 6xmyc tag in blue, and 12xPP7 in dark blue. The stop codon following the GFP tag is marked with a triangle.
(JPG)

S2 Fig Expression and localization of *slam* RNA and protein in blastoderm embryos. **(A)** Relative abundance of *slam* RNA determined by reverse transcription and quantitative PCR, with RNA isolated from embryo collections staged by time after egg lay. Corresponding nuclear cycles are indicated. **(B)** Abundance of Slam protein analyzed by western blot with extracts from heat fixed embryos staged by morphology and nuclear cycle. Detection of α-tubulin serves as a loading control. Corresponding absolute time after fertilization is indicated. **(C)** Wild-type embryos were fixed and stained for *slam* RNA by fluorescent RNA in situ hybridization (grey/red), for Slam protein by immunostaining (grey/green), and for DNA (blue). Images were recorded from 1 slide in 1 session, with the unchanged settings of the confocal microscope. Embryos were staged by the length of the furrow and nuclear density and morphology. Arrows in yellow point to punctate RNA staining inside the nucleus, which likely represent sites of primary transcription. **(D)** Expression of *slamPP7* RNA in embryos with zygotic expression of *slamPP7* in a wild-type background. RNA was detected with a probe specific for the PP7 sequence by fluorescent RNA in situ hybridization. For staging, embryos were stained for Slam protein (red) and DNA (blue), shown in side view. Scheme of the genomic *slamPP7* transgenic construct is shown above the images. Scale bar = 10 μm. C, late cellularization (cycle 14); G, early gastrulation (stage 7); MW, apparent molecular weight; P, preblastoderm (nuclear cycle 1–9).
(JPG)

S3 Fig Characterization of embryos injected with cycloheximide. Images from time-lapse recordings of embryos expressing GFP-slam from a genomic transgene prior to and 10 min and 20 min after injection. Embryos were injected with either buffer or cycloheximide (1 mg/ml). Embryos were injected **(A)** in mitosis 13, **(B)** at the onset of cellularization, and **(C)** in early cellularization. Scale bar = 10 μm.
(JPG)

S4 Fig Mapping of parts of the *slam* RNA sufficient for localization in wild-type embryos. **(A)** Wild-type embryos or *slam* deficient embryos from germ line clones were injected with indicated mRNA and fixed and stained for Slam protein using Slam or Myc antibodies and RNA by probes for *slam* or GFP. *slam* embryos were recognized by the absence of overall *slam* RNA or protein signal. Scale bar = 10 μm. **(B)** Schematic representation of constructs. Detected FC localization of the injected RNA is indicated by “+” and red color of the construct and non-FC localization by “–”and faint red color of the construct. *slam* coding sequence is marked in red, 6xmyc tag in blue, and GFP in green. Dashed lines indicate deletions. Boxes in yellow mark the mapped regions, Loc1, Loc2, Loc3.
(JPG)

S5 Fig Sequences of *slamACU* allele. Comparison of coding sequences of wild-type (capital letters) and ACU (small letters) alleles. Sequences changes in ACU are marked in red. The exon-exon junction of the second intron is marked in yellow. cDNA clone LD22808 contains a third small intron (marked in blue), which is not annotated in the current genome annotation. This piece of sequence was not mutated in the ACU allele.
(PDF)

S6 Fig Expression of the *slam* in cultured S2 cells. **(A, B)** Western blot probed with Slam and α-tubulin antibodies with extracts from cultured S2 cells transiently transfected for expression for *slam*, *slam*[ACU], or GFP. * marks a cross-reacting band that served as loading control. **(C)** Abundance of *slam* transcripts and Slam protein expressed from *slam*[ACU] in relation to *slam*[wild type] was determined by western blotting (protein) and reverse transcription with qPCR (RNA) in extracts of transiently transfected S2 cells. Error bar indicates standard error of the mean. *N* = 3, 3 biological replicates. The underlying data for this figure can be found in S1 Data. ACU, alternative codon usage; GFP, green fluorescent protein; kD, kilodalton; MW, molecular weight; qPCR, quantitative polymerase chain reaction; S2 cells, Drosophila melanogaster Schneider 2 cells.
(JPG)

S7 Fig Phenotype of *slamACU* embryos. The length of the cellularization furrow was measured from time-lapse recordings with differential interference contrast of wild-type embryos and embryos from females with *slam* germ line clones with or without the *slam[ACU]* genomic transgene. Embryos were grouped into mutant (red) and zygotically rescued (blue) according to the cellularization phenotype. Bars = standard error of the mean. The underlying data for this figure can be found in S1 Data. ACU, alternative codon usage; *N*, number of embryos; *slam*, *slow as molasses*.
(JPG)

S1 Table Transcripts specifically associated with Slam protein. (PDF)

S1 Movie Injected fluorescent *slam* RNA associates with FC. Rhodamine-labelled *slam* mRNA was injected into wild-type embryos. Images were recorded every 20 s. The movie starts in interphase 13. During mitosis, the furrow extends and retracts. During interphase 14, the furrow gradually elongates with the FC moving inwards.
(AVI)

S1 Data Source data for the charts shown in Figs 1, 2, 5 and 7 and S2, S6 and S7 Figs. (XLSX)

## Abbreviations

ACU: alternative codon usage
CDS: coding DNA sequence
cncRNA: RNA with coding and noncoding functions
CS: vector plasmid CS2
CyHx: cycloheximide
FC: furrow canal
FRAP: fluorescence recovery after photobleaching
Gal4: galactose-responsive transcription factor
GFP: green fluorescent protein
kD: kilodalton
MW: molecular weight
PCP: bacteriophage PP7 coat protein
PP7: bacteriophage PP7
qPCR: quantitative polymerase chain reaction
S2 cells: *Drosophila melanogaster* Schneider 2 cells
*slam*: *slow as molasses*
TEV: Tobacco Etch Virus nuclear-inclusion-a endopeptidase
UAS: Upstream Activation Sequence
UTR: untranslated region
WT: wild-type
