# Supplementary figures and images for: Slam protein dictates subcellular localization and translation of its own mRNA
Source: PLoS Biol. 2017 Dec 4;15(12):e2003315. doi: 10.1371/journal.pbio.2003315 (PMC5730382; doi:10.1371/journal.pbio.2003315)

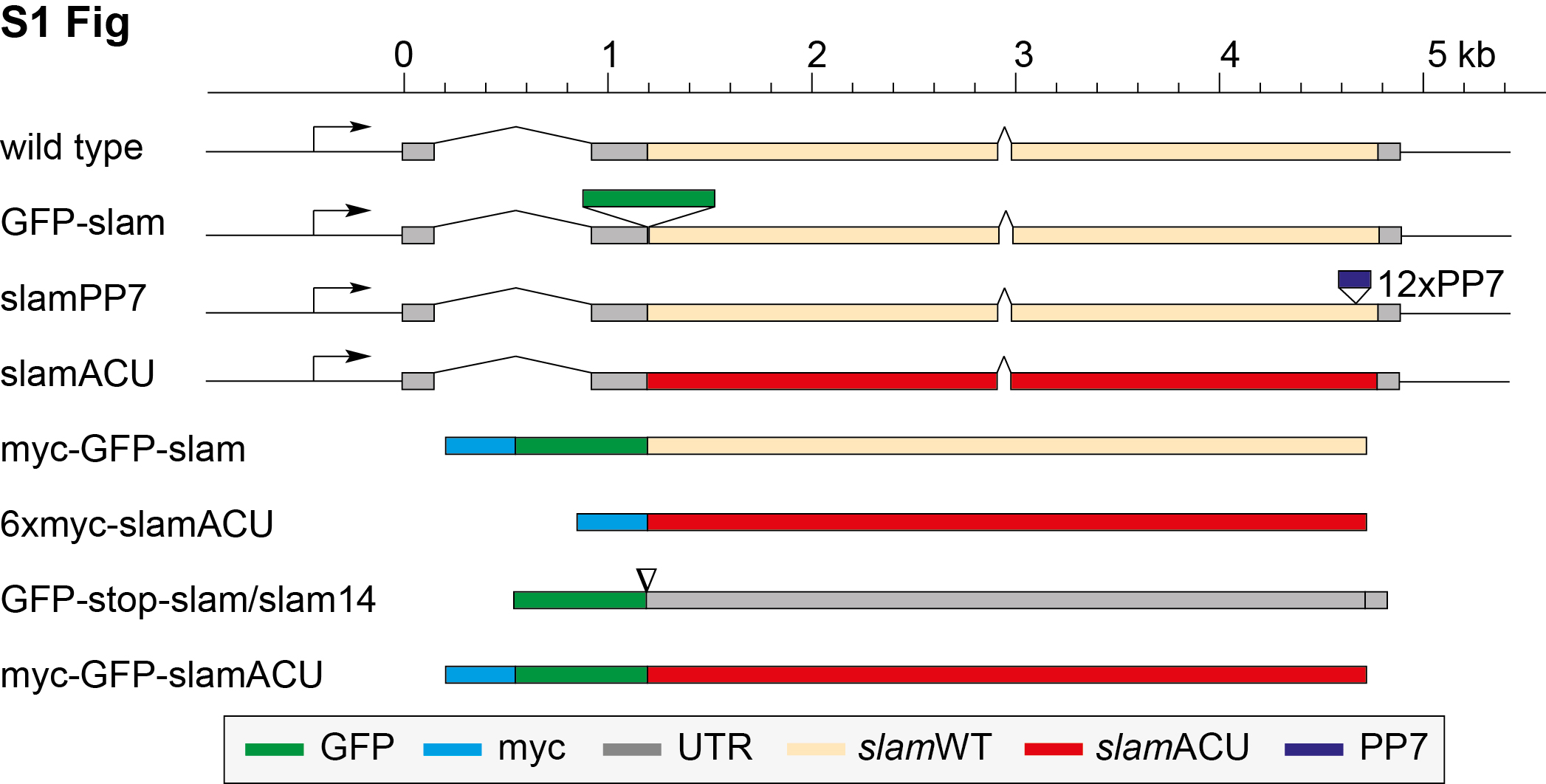

Supplement: S1 Fig — Schematic drawing of genomic slam transgenes and slam cDNA constructs in comparison to the slam locus. Boxes indicate transcribed regions. Coding sequence is marked in light orange, sequences with alternative coding in red, untranslated region in grey, GFP in green, 6xmyc tag in blue, and 12xPP7 in dark blue. The stop codon following the GFP tag is marked with a triangle. (JPG) [file pbio.2003315.s001.jpg]

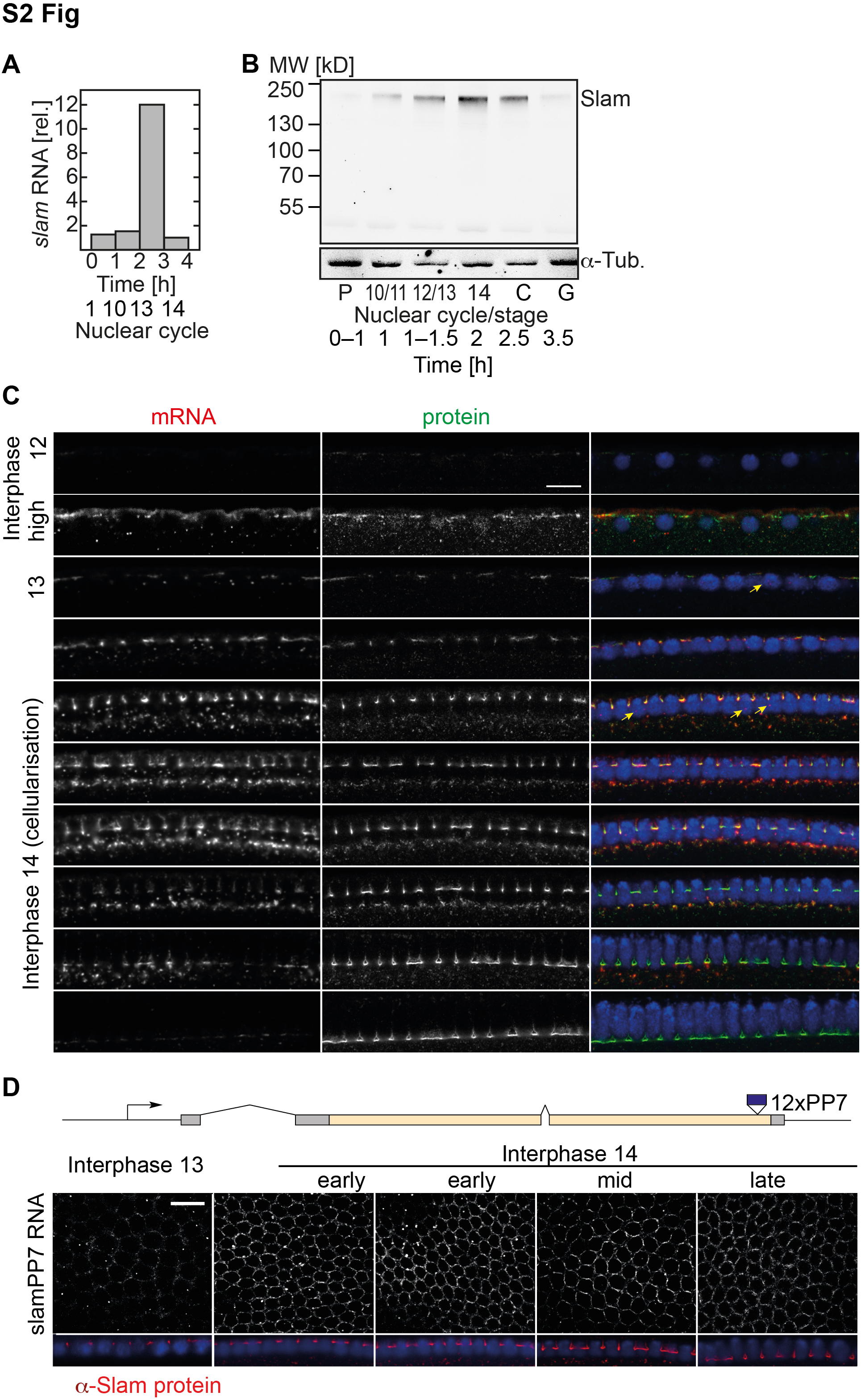

Supplement: S2 Fig — (A) Relative abundance of slam RNA determined by reverse transcription and quantitative PCR, with RNA isolated from embryo collections staged by time after egg lay. Corresponding nuclear cycles are indicated. (B) Abundance of Slam protein analyzed by western blot with extracts from heat fixed embryos staged by morphology and nuclear cycle. Detection of α-tubulin serves as a loading control. Corresponding absolute time after fertilization is indicated. (C) Wild-type embryos were fixed and stained for slam RNA by fluorescent RNA in situ hybridization (grey/red), for Slam protein by immunostaining (grey/green), and for DNA (blue). Images were recorded from 1 slide in 1 session, with the unchanged settings of the confocal microscope. Embryos were staged by the length of the furrow and nuclear density and morphology. Arrows in yellow point to punctate RNA staining inside the nucleus, which likely represent sites of primary transcription. (D) Expression of slamPP7 RNA in embryos with zygotic expression of slamPP7 in a wild-type background. RNA was detected with a probe specific for the PP7 sequence by fluorescent RNA in situ hybridization. For staging, embryos were stained for Slam protein (red) and DNA (blue), shown in side view. Scheme of the genomic slamPP7 transgenic construct is shown above the images. Scale bar = 10 μm. C, late cellularization (cycle 14); G, early gastrulation (stage 7); MW, apparent molecular weight; P, preblastoderm (nuclear cycle 1–9). (JPG) [file pbio.2003315.s002.jpg]

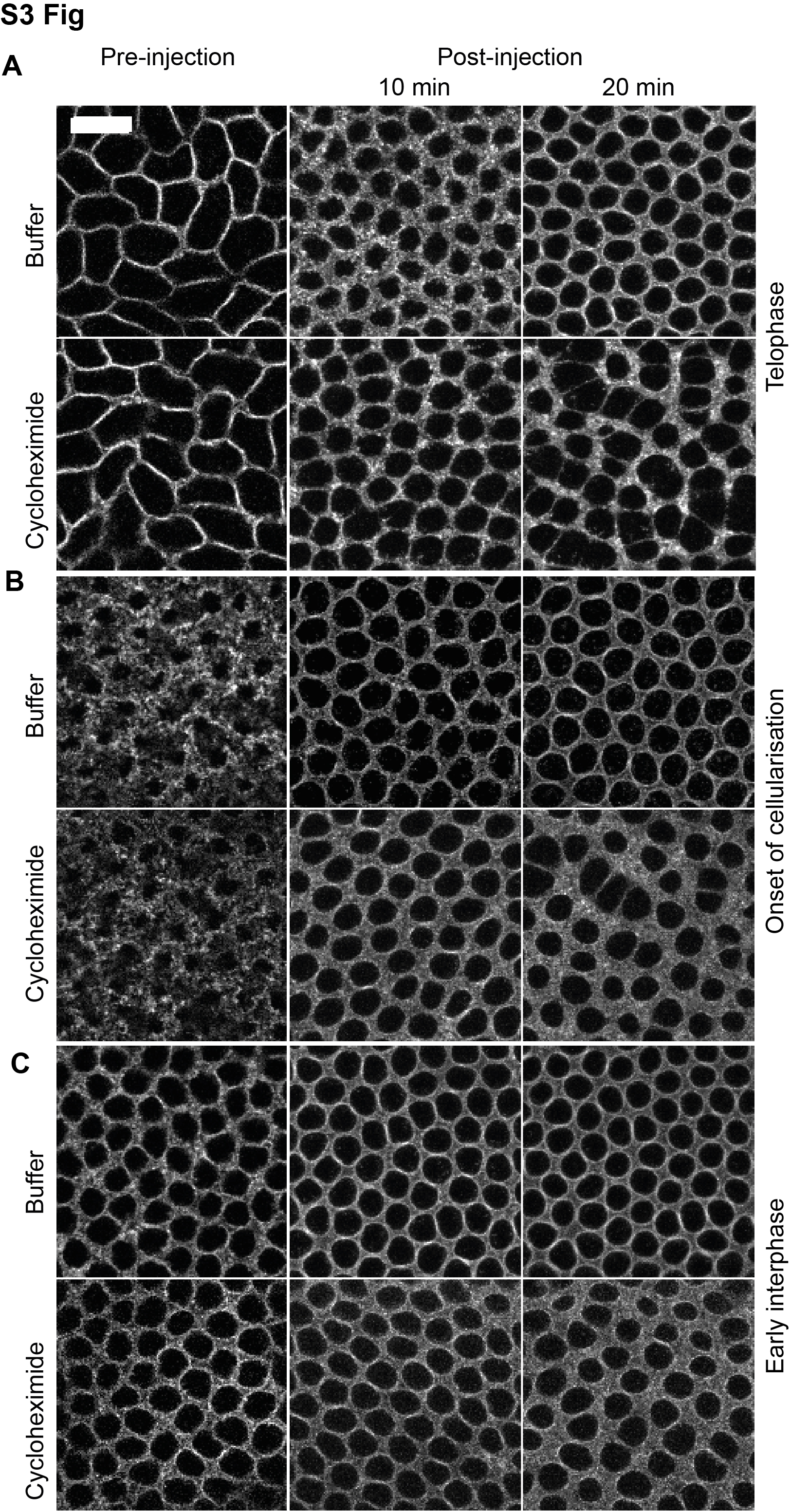

Supplement: S3 Fig — Images from time-lapse recordings of embryos expressing GFP-slam from a genomic transgene prior to and 10 min and 20 min after injection. Embryos were injected with either buffer or cycloheximide (1 mg/ml). Embryos were injected (A) in mitosis 13, (B) at the onset of cellularization, and (C) in early cellularization. Scale bar = 10 μm. (JPG) [file pbio.2003315.s003.jpg]

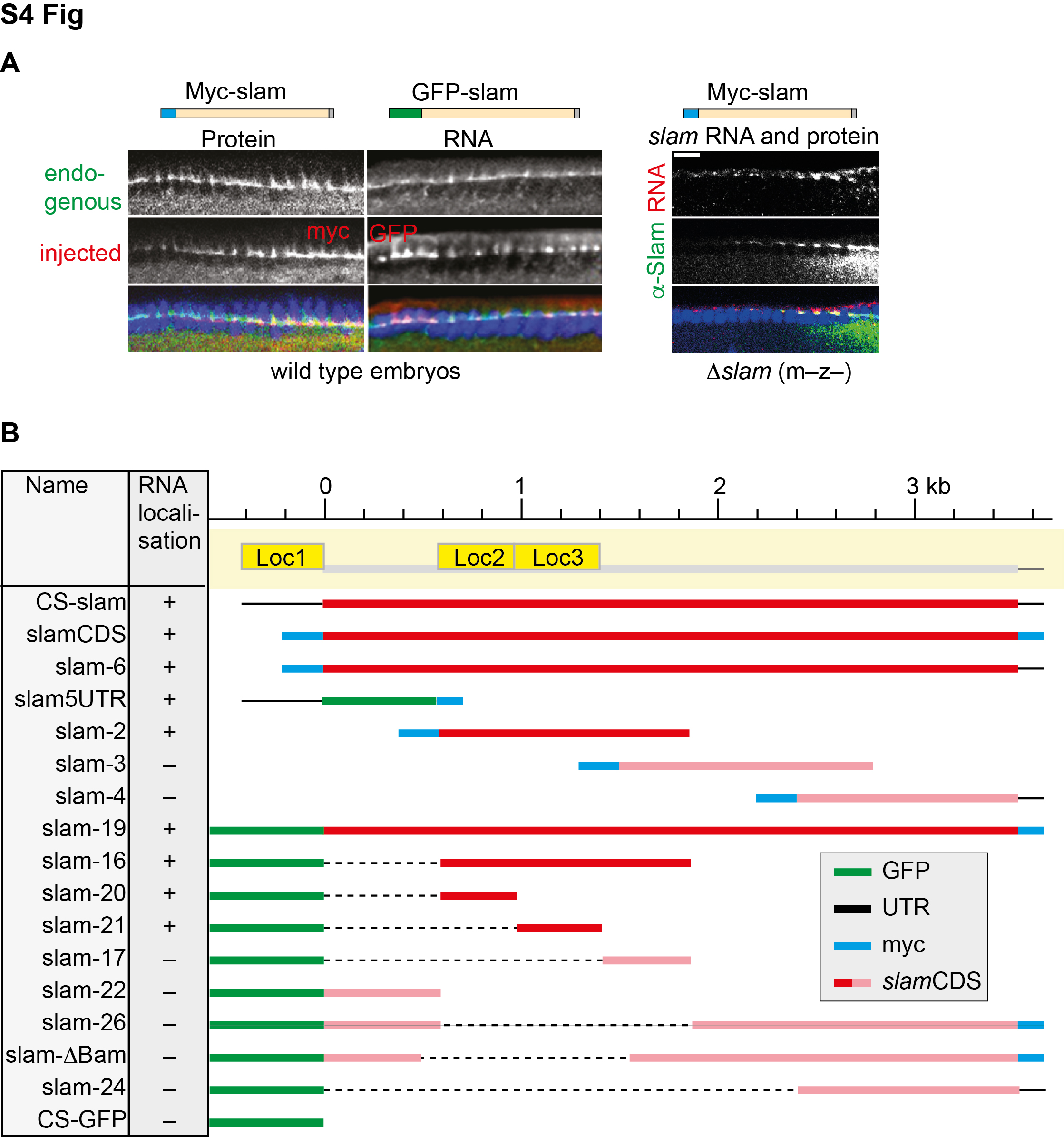

Supplement: S4 Fig — (A) Wild-type embryos or slam deficient embryos from germ line clones were injected with indicated mRNA and fixed and stained for Slam protein using Slam or Myc antibodies and RNA by probes for slam or GFP. slam embryos were recognized by the absence of overall slam RNA or protein signal. Scale bar = 10 μm. (B) Schematic representation of constructs. Detected FC localization of the injected RNA is indicated by “+” and red color of the construct and non-FC localization by “–”and faint red color of the construct. slam coding sequence is marked in red, 6xmyc tag in blue, and GFP in green. Dashed lines indicate deletions. Boxes in yellow mark the mapped regions, Loc1, Loc2, Loc3. (JPG) [file pbio.2003315.s004.jpg]

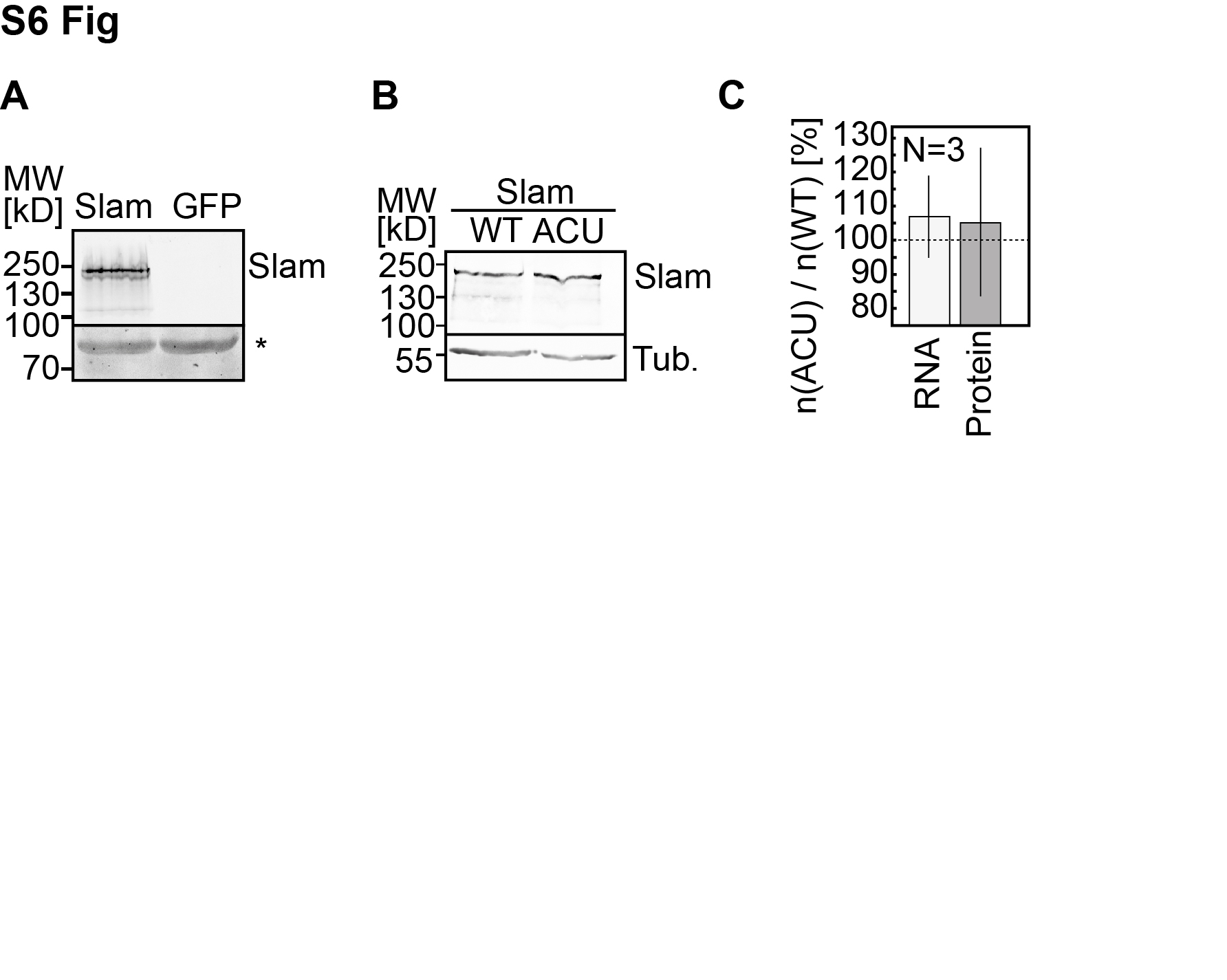

Supplement: S6 Fig — (A, B) Western blot probed with Slam and α-tubulin antibodies with extracts from cultured S2 cells transiently transfected for expression for slam, slam[ACU], or GFP. * marks a cross-reacting band that served as loading control. (C) Abundance of slam transcripts and Slam protein expressed from slam[ACU] in relation to slam[wild type] was determined by western blotting (protein) and reverse transcription with qPCR (RNA) in extracts of transiently transfected S2 cells. Error bar indicates standard error of the mean. N = 3, 3 biological replicates. The underlying data for this figure can be found in S1 Data. ACU, alternative codon usage; GFP, green fluorescent protein; kD, kilodalton; MW, molecular weight; qPCR, quantitative polymerase chain reaction; S2 cells, Drosophila melanogaster Schneider 2 cells. (JPG) [file pbio.2003315.s006.jpg]

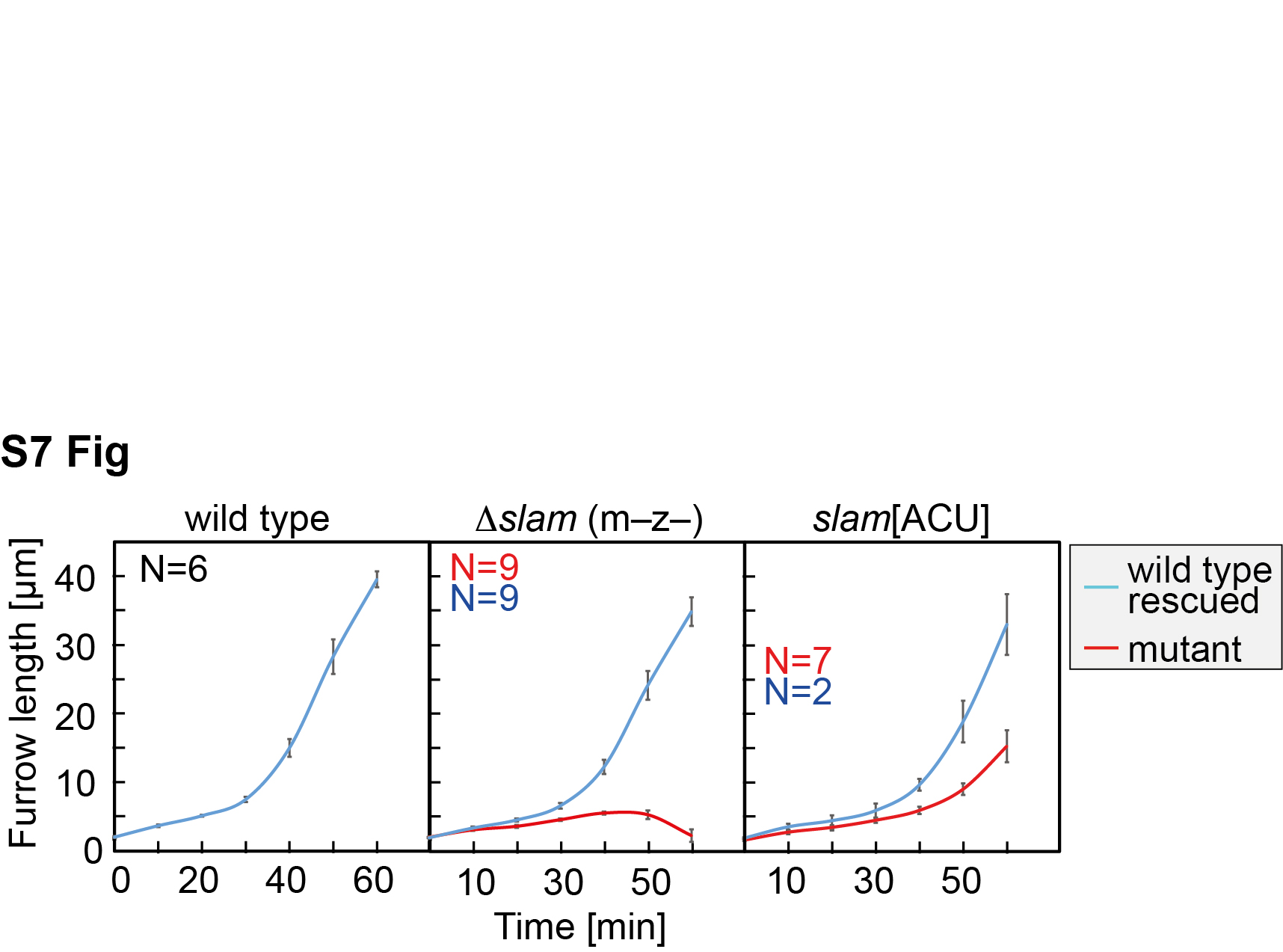

Supplement: S7 Fig — The length of the cellularization furrow was measured from time-lapse recordings with differential interference contrast of wild-type embryos and embryos from females with slam germ line clones with or without the slam[ACU] genomic transgene. Embryos were grouped into mutant (red) and zygotically rescued (blue) according to the cellularization phenotype. Bars = standard error of the mean. The underlying data for this figure can be found in S1 Data. ACU, alternative codon usage; N, number of embryos; slam, slow as molasses. (JPG) [file pbio.2003315.s007.jpg]
