# Supplementary material for: Slam protein dictates subcellular localization and translation of its own mRNA
Source: PLoS Biol. 2017 Dec 4;15(12):e2003315. doi: 10.1371/journal.pbio.2003315 (PMC5730382; doi:10.1371/journal.pbio.2003315)
Supplement: S5 Fig — Comparison of coding sequences of wild-type (capital letters) and ACU (small letters) alleles. Sequences changes in ACU are marked in red. The exon-exon junction of the second intron is marked in yellow. cDNA clone LD22808 contains a third small intron (marked in blue), which is not annotated in the current genome annotation. This piece of sequence was not mutated in the ACU allele. (PDF) [file pbio.2003315.s005.pdf]

Supplemental data. Figure S5. Nucleotide sequence of the slam[ACU] allele (small letters) in comparision to the wild type allele (capital letters). Sequence variations are marked in red.

```
ATG CCA GAA AGC CAC AGT TAC AAA CTG AAA CGT CTA ACG TCA ACA ACA AGA CAG CAA ACT
atg ccc gag tcc cat agc tat aag ttg aag cgc ctg acc tcc acc acg cgc caa cag aca  60
 M   P   E   S   H   S   Y   K   L   K   R   L   T   S   T   T   R   Q   Q   T   20

AAT ACC AAA ATG GTC CTA AGC AAT TCC ACG CCG AAC AAC CAG TCC AAG CAC AAC CAG ATG
aac acg aag atg gtg ctg tcc aac agc acc cca aat aat caa agc aaa cat aat caa atg 120
 N   T   K   M   V   L   S   N   S   T   P   N   N   Q   S   K   H   N   Q   M   40

GTA GTG GAC ACG GCA GCC ATG AAC AGT GAT GAT CTC AGC GAG CTA CTG CAG CTG AAC GCG
gtg gtc gat acc gcc gcg atg aat agc gac gac ctg tcc gaa ctt ctc caa ttg aat gcc 180
 V   V   D   T   A   A   M   N   S   D   D   L   S   E   L   L   Q   L   N   A   60

GAG ATC GAG GAA CGT CGT CGC TCC AGC CGC CAC GGA GAT GCC AGC ACC GCC TGT GGC CTG
gaa att gaa gag cgc cgc cgt agc tcc cgt cat ggc gac gcg tcc acg gcg tgc gga ttg 240
 E   I   E   E   R   R   R   S   S   R   H   G   D   A   S   T   A   C   G   L   80

CTC CGA GCT ACG ATG ACC AGG GAG GAA CTC TTC GAG ATT TCC TCG CTG GAT GAT GAT CGC
ctg cgc gcc acc atg acg cgc gaa gag ctg ttt gaa atc agc tcc ttg gac gac gac cgt 300
 L   R   A   T   M   T   R   E   E   L   F   E   I   S   S   L   D   D   D   R  100

TTC CTG ACC GCC CTG GAG TAC CAA AAC TCG TTT GCA TCA CCG CGC CGA GTG CAG GTT ACC
ttt ttg acg gcg ctc gaa tat cag aat agc ttc gcc agc ccc cgt cgc gtc caa gtg acg 360
 F   L   T   A   L   E   Y   Q   N   S   F   A   S   P   R   R   V   Q   V   T  120

GAC CTG GAC TTG TCC AGC ATC GAG AAT CTG ATG AAG TAC TTC GAC GAA GAG GTA CCT GTG
gat ttg gat ctg agc tcc att gaa aac ttg atg aaa tat ttt gat gag gaa gtg ccc gtc 420
 D   L   D   L   S   S   I   E   N   L   M   K   Y   F   D   E   E   V   P   V  140

ACG CCA ACA AAG ACA CTG GGT ACC ACC AAG GCA GCG GGT AAC ACG GGT AAA GTG GCC AGC
acc ccc acc aaa acc ttg gga acg acg aaa gcc gct ggc aat acc gga aag gtc gcg tcc 480
 T   P   T   K   T   L   G   T   T   K   A   A   G   N   T   G   K   V   A   S  160

ACA ATT GCC AAG TTG GCC CTC CAG TCG GAT CCG GTC ACG CCG CCC AAG CCA AAA GTG GGT
acc atc gcg aaa ctg gcg ctg caa tcg gat ccg gtg acc ccc ccg aaa ccc aag gtc gga 540
 T   I   A   K   L   A   L   Q   S   D   P   V   T   P   P   K   P   K   V   G  180

AGT GGC CAC CTA AAG ATC AGC GAG CTC AAG CAG AAA TAC GAA CAG CTG CCG GAG ATG GAG
agc gga cat ctg aaa att agt gaa ctg aaa caa aag tat gag caa ctc ccc gaa atg gaa 600
 S   G   H   L   K   I   S   E   L   K   Q   K   Y   E   Q   L   P   E   M   E  200

ACG CCA CGC TCT GCA TAT CAG GCT TCC AGA AAG GTA TCC GCG TCG CTG CCC ATG AAG GTC
acc ccg cgg tcc gcc tac caa gcc tcg cgc aaa gtg tcg gcc tcc ctc ccg atg aaa gtg 660
 T   P   R   S   A   Y   Q   A   S   R   K   V   S   A   S   L   P   M   K   V  220

AAG GAG ATG GCC CAG CTG TTT AAC TCC AAG ATC AGC CAG GTG ATG CGA CGT ACA GAG GAA
aaa gaa atg gcg caa ctc ttc aat tcg aaa att agt caa gtc atg cgc cgc acc gaa gag 720
 K   E   M   A   Q   L   F   N   S   K   I   S   Q   V   M   R   R   T   E   E  240

CCA CAG TAT GTT CAG CTG CAA AAT GAG ATG TCG CCG GAG GTT AAG GCA CAG AAC CGC CTG
ccc caa tac gtg caa ctc cag aac gaa atg tcc ccc gaa gtg aaa gcc caa aat cgt ctc 780
 P   Q   Y   V   Q   L   Q   N   E   M   S   P   E   V   K   A   Q   N   R   L  260

GTG TCG CCA TTA GAG TCC CCA GTT CAA GGA CCC TGC CTG GTG GCC GAG GAA GTT TTC CGC
gtc tcc ccg ttg gaa tcg ccc gtg cag ggc cca tgt ctc gtc gct gaa gag gtg ttt cgt 840
 V   S   P   L   E   S   P   V   Q   G   P   C   L   V   A   E   E   V   F   R  280
```

```
GAG TTG AGC GTG AAG GAC AAA GCC CTG CTG TTC AAC AAG TTC ATC GGT GAC ATG GCA GCC
gaa ctg agt gtc aaa gat aag gcg ctc ctc ttt aat aaa ttt att ggc gat atg gcc gct 900
 E   L   S   V   K   D   K   A   L   L   F   N   K   F   I   G   D   M   A   A  300

AAG CAC CCC AAG TTC ACT GCA CAT GCC GCA GAT CTC AAG GAG AAG GTA AAC AAA CAG GTG
aaa cat ccg aaa ttt acc gcc cac gca gcc gac ctg aaa gaa aaa gtg aat aag caa gtc 960
 K   H   P   K   F   T   A   H   A   A   D   L   K   E   K   V   N   K   Q   V  320

GCA CGA GGG GAG GTG GTG GCT GAA CGA CAA GCC AGT GTT AAG CAT CTC GCC CAG GAG TTG
gcc cgc ggc gaa gtc gtc gcc gag cgc cag gcg agc gtg aaa cac ctg gct caa gaa ctg 1020
 A   R   G   E   V   V   A   E   R   Q   A   S   V   K   H   L   A   Q   E   L  340

GAA GCG AAA TGT ATC CTG GAG CCG GGA TCG CCA CCG CGA CCA GGT GGT GTC TCT CCC CCC
gag gcc aag tgc att ctc gaa ccc ggc tcc ccg cca cgc ccc ggc ggc gtg tcc ccg ccg 1080
 E   A   K   C   I   L   E   P   G   S   P   P   R   P   G   G   V   S   P   P  360

AAG ACG ACT GAG AGC AAA ACG GAG ACA TGC ACC TCC CAG CTG CAT GTG AGC ACC CTG ACT
aaa acg act gag agc aaa acg gag aca tgc acc tcc cag ctg cat gtg agc acc ctg act
 K   T   T   E   S   K   T   E   T   C   T   S   Q   L   H   V   S   T   L   T

GTG ATC CTC AAG
gtg atc ctc aag
 V   I   L   K

    CCA AGT CCA GAG CGC AGA GCT CCA CAG CCA CGA CCC CGC CGA TGC CTG GAT CGT CAG
    ccc agc ccc gaa cgt agg gcc ccg caa ccg cgc cca cga cgc tgt ctc gac cgc caa 1140
     P   S   P   E   R   R   A   P   Q   P   R   P   R   R   C   L   D   R   Q  380

AGC GAT GAA CAT GCC CGA GAG CCA TCC CAG AAG CGA AAC CTC GCC GCG ATT CGC ACC ATG
agt gac gag cac gct cgc gaa ccc tcg caa aaa cgc aat ctg gct gcc atc cga acg atg 1200
 S   D   E   H   A   R   E   P   S   Q   K   R   N   L   A   A   I   R   T   M  400

ATG CCA ACG GAG GCC TAT GCG CCA CCA AAG AAA ATC CGA CGC ACT CGA CAG GAG CGC ACC
atg ccc acc gaa gct tac gcc ccg ccg aaa aag att cgc cga acc cgc caa gaa cga acg 1260
 M   P   T   E   A   Y   A   P   P   K   K   I   R   R   T   R   Q   E   R   T  420

GGT GCG GAT AGC CAA ATG TTT TTT CAG AAC GAA CAC CTC GAA ACC CTG TTC TAC AGC TGG
ggc gcc gac agt cag atg ttc ttc caa aat gag cat ctg gag acg ctc ttt tat agt tgg 1320
 G   A   D   S   Q   M   F   F   Q   N   E   H   L   E   T   L   F   Y   S   W  440

CTC AGC TCG GAG AAC GGT GTC CAG TTC GAC ATT ACG AGT GTG TCT GAC GGT CAG CAG ACC
ctg agt tcc gaa aat ggc gtg caa ttt gat atc acc agc gtc tcg gat ggc caa caa acg 1380
 L   S   S   E   N   G   V   Q   F   D   I   T   S   V   S   D   G   Q   Q   T  460

ATT GAG ATA GCC ACC GAG GAG GGT AAT CTT CTG GAG CAA CCG CTA CTT GAG CCG AGC ACC
atc gaa att gct acg gaa gaa ggc aac ctg ctt gaa cag ccc ctt cta gaa ccc agt acg 1440
 I   E   I   A   T   E   E   G   N   L   L   E   Q   P   L   L   E   P   S   T  480

GCT ACT CTG GAG GAT GTG AGC CAA AAG TCC GCC GTC GAG CGG CTT CTG GAG GAG GCC ATC
gcc acc ctc gaa gac gtc agt cag aaa tcg gct gtg gaa cgc ctg ctc gaa gaa gcg att 1500
 A   T   L   E   D   V   S   Q   K   S   A   V   E   R   L   L   E   E   A   I  500

GCA AAA TTA GAG CTA GAT AAC GAG TCC AAG AAG GAG ACA CAA GTC GAG GAA AAG AAG GAG
gcc aag ttg gaa ctg gac aat gaa tcg aaa aaa gaa acc cag gtg gaa gag aaa aaa gaa 1560
 A   K   L   E   L   D   N   E   S   K   K   E   T   Q   V   E   E   K   K   E  520

ATG GAT ACA GCT CAG GAT CCC ATC GTT CAA GTC ACT CCG CGA CGC ATT AAG CGC CAG GCG
atg gac acc gcc cag gat ccg att gtg cag gtg acc ccc cgc cgt atc aaa cgt caa gcc 1620
 M   D   T   A   Q   D   P   I   V   Q   V   T   P   R   R   I   K   R   Q   A  540
```

```
CCG CCA GTG CCA GCC CCA CGA CCC AGT CTA AGT CAA GTT ACC TCC AGT GCA TCC AGC TGC
ccc ccg gtc ccc gct ccg cgc ccg agc ttg agc cag gtg acg agc tcc gct agc tcc tgt 1680
 P   P   V   P   A   P   R   P   S   L   S   Q   V   T   S   S   A   S   S   C  560

AAG CAA TCC GAG GCT GAG GAA AGT GAA TCC GGG TTG TCT ACA CTC CCC AAG|ATA ACC AGC
aaa cag agc gaa gcc gaa gag agc gag agc ggc ctg agc acc ctg ccc aag|ata acc agt 1740
 K   Q   S   E   A   E   E   S   E   S   G   L   S   T   L   P   K   I   T   S  580

GAC GAA TCC CAG CCA GAG ACG CCC AAG GAT GAC TTG CAG ACA GGT GAA TTT GAT TTT GCC
gat gag agc caa ccg gaa acc ccg aaa gac gat ctg caa acc ggc gag ttc gac ttc gcg 1800
 D   E   S   Q   P   E   T   P   K   D   D   L   Q   T   G   E   F   D   F   A  600

AAG CCC CAG CGT CCG CCA CGC AAG AAG AAA ATG CGC CGA ACT CTT ACA TGG AAA AAG GAG
aaa ccg caa cgc cca ccg cgt aaa aaa aag atg cgt cgc acc ctg acc tgg aag aaa gaa 1860
 K   P   Q   R   P   P   R   K   K   K   M   R   R   T   L   T   W   K   K   E  620

AAC TCC ATT GTC GAG GCC ACT GCA ATA ACC TCA ACG GAC TCC GAT TCA GAT TAC AAG CCT
aat agc atc gtg gaa gct acc gcc atc acg tcc acc gat agc gac tcc gac tat aaa ccg 1920
 N   S   I   V   E   A   T   A   I   T   S   T   D   S   D   S   D   Y   K   P  640

CCG CTA TCA GGA GCG GCC AAG AAG AAG TCC GGT GCT ATC TGT AAC CCA CTT CCA ATG CCC
ccc ctc agt ggc gcc gcg aaa aag aaa agc gga gcc att tgc aat ccg ctg ccg atg cca 1980
 P   L   S   G   A   A   K   K   K   S   G   A   I   C   N   P   L   P   M   P  660

GAG CCA AGT TTC ATT GAG TTG GAT AAG TCA CTG ATG CGC CAC TTG AAC TCG CCC CGG AAG
gaa ccg agc ttt atc gaa ctg gac aaa tcc ttg atg cgt cat ctg aat tcc ccg cgc aaa 2040
 E   P   S   F   I   E   L   D   K   S   L   M   R   H   L   N   S   P   R   K  680

ATC AAG TCG GCA TAC ACC CTC ACC GTG ATG TCA TCT CCC TCG CCG AAT GCA GAC AGC GAT
att aaa agc gcc tat acg ctg acg gtc atg agc tcc ccg tcc cca aac gcc gat tcc gac 2100
 I   K   S   A   Y   T   L   T   V   M   S   S   P   S   P   N   A   D   S   D  700

CAG TCT CCT AGT CAG ACA CCA AGG CAA TCC CTG GTA CAG GCC ATG CGG GAC AGT TTC GTG
caa tcc ccg agc caa acc ccg cgc cag agc ttg gtg caa gcg atg cgc gat agc ttt gtc 2160
 Q   S   P   S   Q   T   P   R   Q   S   L   V   Q   A   M   R   D   S   F   V  720

GAT CAG GGC TTT GAG ACA TGC TCG AAC GAT CCG ATG GAC AAC AGT CCG ATA CGC CGA TCT
gac caa gga ttc gaa acc tgt tcc aat gac ccc atg gat aat agc cca att cgt cgt tcc 2220
 D   Q   G   F   E   T   C   S   N   D   P   M   D   N   S   P   I   R   R   S  740

TCG CTT GGA GCC ACG GAA TCG AAG CCA TCC GGT TTT TCC ACG CCA GTT AAG GGA CGC CAT
agc ctg ggt gcg acc gag agc aaa ccg agc ggc ttc tcg acg ccc gtg aaa ggc cgt cac 2280
 S   L   G   A   T   E   S   K   P   S   G   F   S   T   P   V   K   G   R   H  760

GCT TCC AGT CCG GCG CAA CAA CAA CTG TTC AGT CCG ATT CTC ATC CAG GAG CGC CCT CGT
gcc agc agc ccc gcc cag cag cag ctc ttt agc cca atc ctg att caa gaa cgt ccg cgc 2340
 A   S   S   P   A   Q   Q   Q   L   F   S   P   I   L   I   Q   E   R   P   R  780

CGC AGT TCC CTG GCC ATG CAA GTG ATC CGG GAG GAT CAT CCG CTC GAT CTG GAC GCC ACC
cgt agc agc ctc gcg atg cag gtc att cgc gaa gac cac ccg ctg gac ttg gat gcg acg 2400
 R   S   S   L   A   M   Q   V   I   R   E   D   H   P   L   D   L   D   A   T  800

TCC AGC TCA CCC TCC ACA CCG TGC AGT GAG CGG GAG TTC TTC GCC AAT GCC CCG ACT GTT
agc tcg tcc ccg tcg acc ccc tgt agc gaa cgc gaa ttt ttt gcg aac gcg ccc aca gtg 2460
 S   S   S   P   S   T   P   C   S   E   R   E   F   F   A   N   A   P   T   V  820

GAA ATT GAT AAC TCC CAG GAT GAG AGT CCG ACC GGC AAG GCG CAC TCA ATG TTC TGG ATC
gag atc gac aat agc caa gac gaa agc ccc acg gga aaa gcc cat tcc atg ttt tgg att 2520
 E   I   D   N   S   Q   D   E   S   P   T   G   K   A   H   S   M   F   W   I  840
```

ACC TCT GGG GAC TTC ACA GTC TCC CTG GAA ATC TTC AAG AAC AGC CCG GAG CGC CTG CGA
acg tcc ggc gat ttt acg gtg agc ttg gag att ttt aaa aat tcc cca gaa cgt ttg cgc 2580
T S G D F T V S L E I F K N S P E R L R 860

CTG CTG TAC GAA ATC TTC ACC CAG AAA AGC TGG GAG ACT CGT GAC CTG GCC TTC GGA ATC
ttg ctc tat gag att ttt acg caa aag tcc tgg gaa acc cgc gat ttg gcg ttt ggt att 2640
L L Y E I F T Q K S W E T R D L A F G I 880

GAC GGA CAC AAG TTC ATT CGC GGA GCT CCC AGT TCC GAC TCC GTC CGA CAG TCG CTG CCC
gat ggc cat aaa ttt atc cgt ggc gcc ccg agc agc gat agc gtg cgc caa tcc ttg cca 2700
D G H K F I R G A P S S D S V R Q S L P 900

GAG CGT CCA CCC AGT GTG AAG GGC TGC TCT CAC TAT TGG TTC GCC AGC GGA GAT CTC GCA
gaa cgc ccg cca agc gtc aaa gga tgt agc cat tac tgg ttt gcg tcc ggc gac ctg gcc 2760
E R P P S V K G C S H Y W F A S G D L A 920

GTG CCC TTC AGC GGA AAA CTG ATG TCC AGC GAG AAG ATC GAG CGG CTG TTT GCC TTC CTC
gtc ccg ttt tcc ggc aag ttg atg agc tcc gaa aaa att gaa cgt ttg ttc gcg ttt ctg 2820
V P F S G K L M S S E K I E R L F A F L 940

AGC GGA GAG CAG TCG GAG TTG CGC TTC GGC GTT GAC CAC ATT GAG TTC AGC AGC GTG CCT
tcc ggc gaa caa agc gaa ctg cgt ttt gga gtc gat cat atc gaa ttt tcc agt gtc ccc 2880
S G E Q S E L R F G V D H I E F S S V P 960

GAG TTC TGG CCC ACC ACC CAG AAG TAT TCC ATC GAG AGC AGC TAC AGC ATA CTG GTG GGT
gaa ttt tgg ccg acg aca caa aaa tac agc att gaa tcc agt tat tcc atc ttg gtc gga 2940
E F W P T T Q K Y S I E S S Y S I L V G 980

CTG CAG ACA GGT GCC AGC AAT GGC CTG GAG GGT CGC AGC AAG TAC TCC TGG CCC AAC AGC
ctc caa acc gga gct tcc aac gga ttg gaa ggc cgt tcc aaa tat agc tgg ccg aat tcc 3000
L Q T G A S N G L E G R S K Y S W P N S 1000

AGC ATC AGT GCC AAC CAG GCG ATC AAG ACC AGC GAC CTG GAT CAG ACG GAG TTC GAG TCC
tcg att agc gct aat caa gcc att aaa acg tcc gat ttg gac caa acc gaa ttt gaa agc 3060
S I S A N Q A I K T S D L D Q T E F E S 1020

GAT AGC TTC GGC AAC AAC AGT GGT AGG CTG TCC TTT TCG CCT GAT CTC TTC TCC CTG GAC
gac tcc ttt gga aat aat agc ggc cgc ttg agc ttc agc cca gac ctg ttt agc ttg gat 3120
D S F G N N S G R L S F S P D L F S L D 1040

TAC GAG GCG GTT CCC CTG GAC GAG CTG TTT GCC AAG GCT CCT CCC TCT GCC GCC ACA CCC
tat gaa gcc gtg ccg ttg gat gaa ttg ttc gct aaa gcc cca ccg agc gcg gcg acc cca 3180
Y E A V P L D E L F A K A P P S A A T P 1060

GCC ATG TCC GTG CCG CAA ATG ATG CAG ACC CTC AAG CAG CAG CAG TCC AAA CTT CGC AGT
gct atg agc gtc cca cag atg atg caa acg ctg aaa caa caa caa agc aag ctg cgt agc 3240
A M S V P Q M M Q T L K Q Q Q S K L R S 1080

GTG GAG CAG CGC ATT CGT GGC TAC GCC AAG CCC GCG AAT CTG GCC GAC TCT TCG CTG GAG
gtc gaa caa cgt atc cgc gga tat gcg aaa ccg gcc aac ttg gcg gat tcc agc ttg gaa 3300
V E Q R I R G Y A K P A N L A D S S L E 1100

CAC TGC CGC AAC ACG CCG CAA TAT GTC CAC AAG CTG CGC TCC ATC ATC CGG GCC ATT GAC
cat tgt cgg aat acc cca cag tac gtg cat aaa ttg cgt agc att att cgc gcg atc gat 3360
H C R N T P Q Y V H K L R S I I R A I D 1120

AAC ATT GGA CGC GAC GAT GGC TTC CGC GGC TGC TCG ATG GAG CAG CTC GAG AGC TTC ATG
aat atc ggc cgt gat gac gga ttt cgt gga tgt tcc atg gaa caa ctg gaa tcc ttt atg 3420
N I G R D D G F R G C S M E Q L E S F M 1140

```
TAC TTC CTC AGC GAG TAT GCG GAT GTG TGC CTG GCC AAC TGC AGC GAG CAC ATG GAC AAG
tat ttt ctg tcc gaa tac gcc gac gtc tgt ttg gcg aat tgt tcc gaa cat atg gat aaa 3480
 Y   F   L   S   E   Y   A   D   V   C   L   A   N   C   S   E   H   M   D   K  1160

ATT CTC GAC ACC CTG ATG GAC CGG AGG GCC GTG GAG GTC TGA
atc ctg gat acg ttg atg gat cgc cgc gct gtc gaa gtg tga                         3522
 I   L   D   T   L   M   D   R   R   A   V   E   V   .                          1173
```

Capital letters: slam wild type
Small letters: slam acu
Red letters: mutations in slam acu
Blue letters: region that is spliced out in the cDNA clone LD22808 but not in other cDNA clones such as LD36405
| Exon-Exon junction
