# Supplementary material for: Slam protein dictates subcellular localization and translation of its own mRNA
Source: PLoS Biol. 2017 Dec 4;15(12):e2003315. doi: 10.1371/journal.pbio.2003315 (PMC5730382; doi:10.1371/journal.pbio.2003315)
Supplement: S1 Table — (PDF) [file pbio.2003315.s008.pdf]

Supplemental data Table S1: Transcripts specifically associated with Slam protein

Gene	Expression # transcript 1.5–2.5h (1)	N (2) slam	N (3) GFP slam
CG14749	4283	6.33	0.06
Hip1	3482	6.24	0.02
slam	72789	6.14	4.02
casp	153	5.61	0.2
CG32365	1006	5.37	-0.22
crn	2729	4.98	-0.05
CG9776	5922	4.88	0.01
CG14894	3573	4.69	0.26
CG5004	478	4.23	-0.04
eIF3-S10	14391	4.22	-0.12
CG12730	138	4.09	-0.34
CG43659	699	3.96	0.39
CdGAPr	1105	3.92	0.21
pigs	151	3.79	0.36
Uhg2	66	3.79	-0.49
CG17931	717	3.77	-0.06
Cep135	1145	3.76	0.09
Ube3a	4063	3.72	0.07
CG43725	2997	3.55	0.22
Uhg4	119	3.55	-0.26
CG42699	815	3.53	-0.32
Uhg8	57	3.42	0.17
CG13454	469	3.35	-0.04
pros	219	3.22	-0.23
CG3964	198	3.21	-0.01
CG43184	6531	3.19	-0.03
CG8369	88	3.17	0.48
CG4440	2972	3.16	-0.06
Mical	373	3.14	-0.51
Ice1	187	3.03	0.32
ZC3H3	761	3.01	-0.51
Uhg5	370	2.98	-1.57
CG1635	396	2.92	0.31
Rtnl1	13977	2.90	-0.07

Gene	Expression # transcript 1.5–2.5h (1)	N (2) slam	N (3) GFP slam
srw	1597	2.89	0.22
CG31313	154	2.86	0.22
RhoGAP93E	1667	2.86	0.14
unc-13	5120	2.82	-0.68
CG3198	1064	2.78	-0.52
GCC185	2037	2.77	0.07
Obp56a	1105	2.76	-0.24
lft	75	2.76	-0.51
CG10732	1830	2.75	0.44
lilli	2364	2.72	0.05
CG13427	10326	2.72	-0.42
Hsromega	259	2.70	-0.34
Cpr60D	142	2.62	0.84
CG13713	1658	2.61	0.55
roX1	8638	2.58	-0.34
CG34383	286	2.56	-0.7
CG14915	6278	2.54	-0.38
Uhg1	259	2.53	0.13
scra	5451	2.53	0.46
CG18428	383	2.51	-0.3
CG30287	85	2.51	-0.08
CG4702	855	2.49	0.3
CG13000	2634	2.49	0.23
CG32104	181	2.48	0.42
comm2	117	2.47	-0.57
Pabp2	3675	2.46	0.1
CG34266	1093	2.46	0.38
Brd	202	2.40	-0.33
Tes	599	2.39	2.17
Ilp4	2858	2.35	-0.05
CG15479	463	2.35	0.41
CR43887	4190	2.33	-0.64
sisA	998	2.32	0.02
flfl	12379	2.32	3.15

Gene	Expression # transcript 1.5–2.5h (1)	N (2) slam	N (3) GFP slam
BobA	304	2.31	-1.51
SNCF	9578	2.31	-0.16
CG4294	755	2.31	-1.76
CG42238	198	2.30	-0.18
Nipped-B	11573	2.28	-0.05
Jhe	387	2.28	-0.51
Ocho	201	2.26	-0.29
MP1	211	2.26	0.32
CG12420	550	2.26	-0.56
CG1806	88	2.26	0.1
eIF3-S9	20640	2.25	0.15
CR43432	122	2.24	-0.52
CG34224	239	2.23	-0.14
CG3625	271	2.22	0.43
skl	632	2.22	-0.56
zen2	632	2.20	0.11
Obp99a	71	2.19	-0.23
CG42762	846	2.19	1.16
Crtc	770	2.19	0.3
CG7358	3024	2.15	0.06
tsg	1653	2.14	0.21
Atx-1	218	2.13	-0.46
HERC2	168	2.13	0.01
Cys	4100	2.12	-1.71
CG3907	364	2.11	-0.33
bou	104	2.11	-0.34
CG8929	4998	2.09	0.04
CG4953	692	2.09	0.08
CG13711	2784	2.09	0.13
CG4266	3835	2.04	0.05
CG4203	2789	2.02	0.05
CG15876	3697	2.02	0.09
CG5815	1071	2.01	0.27
Patj	1999	2.00	0.00

Gene	Expression # transcript 1.5–2.5h (1)	N (2) slam	N (3) GFP slam
CG18547	441	0.04	6.77
CG12560	212	1.71	5.98
GstD1	55	-0.2	5.30
CG5455	194	0.26	4.34
GstD9	568	0.88	4.29
slam	72789	6.14	4.02
Arc2	141	-0.68	3.29
flfl	12379	2.32	3.15
cindr	83	1.02	2.67
DNaseII	127	0.73	2.45
CG14688	1075	0.17	2.44
CG7381	496	0.2	2.41
Sp7	304.	-0.16	2.28
Tes	599	2.39	2.17
CG5261	441	-0.07	2.12
CG10877	825	-1.19	2.08
**Highly abundant transcripts**			
Ef1alpha48D	295136	-0.95	-0.01
EF2	153904	-0.26	-0.24
Act5C	105667	-0.61	-0.2
betaTub56D	96965	-1.01	-0.25
Df31	95694	-0.46	-0.21
RpS3A	93964	-0.52	-0.2
lola	78336	-0.42	-0.01
alphaTub84	77829	-0.66	-0.2
kuk	76855	-0.62	0.02
slam	72789	6.14	4.02
RpL5	69908	0.02	-0.04
RpL10	67130	-0.8	-0.12
Tctp	66348	-0.59	-0.01
RpL3	62563	-0.09	0.04
RpL7A	59851	-0.79	-0.18
RpS4	59793	-0.46	-0.04

(1) Transcript abundance in 1.5–2.5h embryos. Data from Winkler et al (2017)

(2) Log2 of difference in transcript reads from immunoprecipitation of wild type lysate with Slam and Dia antibodies. Cut off: abundance higher than 50 transcripts

(3) Log2 of difference in transcript reads from immunoprecipitation with GFPbinder from GFPslam and wild type lysate. Cut off: abundance higher than 10 transcripts
